# Redox Homeostasis within the Drug-Resistant Malarial Parasite Digestive Vacuole

**DOI:** 10.1021/acs.biochem.4c00750

**Published:** 2025-05-01

**Authors:** Andreas Willems, Therese Oertel, Paul D. Roepe

**Affiliations:** Depts. of Chemistry and of Biochemistry and Cellular and Molecular Biology, Georgetown University, 37th and O Streets NW, Washington, District of Columbia 20057, United States

## Abstract

We have developed a cost-effective strategy for the complete synthesis of azetidinyl coumarin fluorophore derivatives that report changes in physiologic levels of glutathione (GSH), which includes a more cost- effective synthesis of the probe precursor hydroxyl derivative and its subsequent derivatization to promote subcellular localization. We functionalize coumarin derivatives with a cyano side chain similar to a previous strategy (Jiang X. et al., *Nature Communications* **2017,** 8; 16087) and validate the 7-azetidinyl conformation as an explanation for enhanced GSH-dependent coumarin fluorescence. We couple the azetidinyl probe to different mass dextrans using either no linker or a 6C linker and also synthesize a morpholino derivative. We titrate the fluorescence of the different functionalized probes vs [GSH] *in vitro*. We load one dextran-conjugated probe within the digestive vacuole (DV) of live intraerythrocytic P. falciparum malarial parasites and also measure cytosolic localization of the morpholino probe. Using significantly improved single-cell photometry (SCP) methods, we show that the morpholino probe faithfully reports [GSH] from the live parasite cytosol, while the 70 kDa dextran-conjugated probe reports DV redox homeostasis for control chloroquine-sensitive (CQS) and artemisinin-sensitive (ARTS) transfectant parasites vs their genetically matched chloroquine-resistant (CQR)/artemisinin-sensitive (CQR/ARTS) and CQR artemisinin-resistant (CQR/ARTR) strains, respectively. We quantify rapid changes in DV redox homeostasis for these parasites ± drug pulses under live-cell perfusion conditions. The results are important for understanding the pharmacology of antimalarial drugs and the molecular mechanisms underlying CQR and ARTR phenomena.

## Graphical abstract

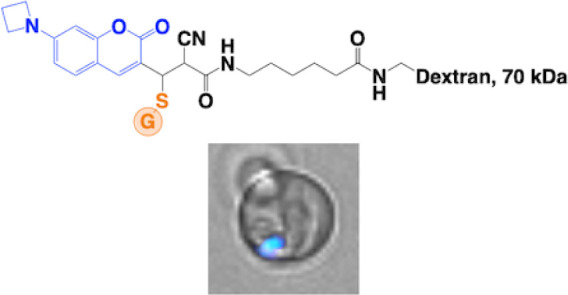

## Introduction

Malaria remains one of the most serious public health threats in the world. Understanding antimalarial drug resistance biochemistry, aimed at facilitating better diagnostics and the design of second-tier therapies, remains a key goal. Currently, artemisinin (ART) combination therapies (ACTs) are the frontline treatments for P. falciparum malaria,^1,2^ but these are beginning to fail in some regions,^3,4^ heightening the need to better understand both the molecular pharmacology of and mechanisms of resistance to the drugs used in various ACTs. Recent work has shown that the activity of ART drugs depends upon reductive cleavage of their endoperoxide bridge, presumably via Fenton chemistry within the parasite, which is easily catalyzed by ferrous protoporphyrin IX heme (Fe­(II) heme, [Fe­(II)­PPIX]) released upon hemoglobin (Hb) catabolism within a specialized lysosome, the intraerythrocytic P. falciparum digestive vacuole (DV).^5–8^ We have shown that dihydroartemisinin (DHA)-FPIX covalent adducts catalyzed by ferrous FPIX are indeed formed within live DHA-treated parasites and that the abundance of these adducts is altered for ARTR vs ARTS P. falciparum.^9,10^ This suggests a possible feature of ARTR parasites, altered redox homeostasis within the DV, which could then influence the ratio of ferrous to ferric FPIX. Since the dominant regulator of intracellular redox potential for eukaryotic cells, including P. falciparum malarial parasites, is glutathione (GSH),^11,12^ such altered redox homeostasis could be the result of perturbed glutathione (GSH) synthesis or transport. As hypothesized by Ginsburg and others,^13^ reduced drug potency for drug-resistant parasites could be connected to such redox homeostasis perturbations. However, no studies to our knowledge have yet been able to probe redox biochemistry within the DV for live drug-sensitive versus drug-resistant parasites upon continuous perfusion ± drug.

In particular, there have been no direct measurements of the concentration of GSH within the DV for live parasites ([GSH]^DV^) nor has there been quantification of any changes in [GSH]^DV^ after drug exposure. Since GSH cycling is the dominant manner in which cells regulate their redox homeostasis, such measurements would directly test long-standing hypotheses that oxidative damage caused by drugs is related to their potency and would test whether DV redox homeostasis is relevant for antimalarial drug pharmacology and resistance.

The DV is a specialized lysosome^14^ whose primary function emerges during the intraerythrocytic stage of parasite development, wherein red blood cell (RBC) hemoglobin (Hb) is trafficked to the DV and degraded by an elegant proteolysis pathway involving both cys and asp proteases.^15–17^ Ultimately, Hb catabolism results in Hb-derived amino acids being used within parasite metabolism and provides adequate intraerythrocytic volume into which the rapidly growing intraerythrocytic parasite can expand.^18,19^ However, Hb catabolism also results in the release of redox-active, toxic ferriprotoporphyrin IX (FPIX) heme. In humans and other higher eukaryotes, when Hb is degraded during RBC turnover,^20^ the FPIX tetrapyrrole is degraded to bile pigments, while Fe is scavenged and recycled. However, lacking the heme oxygenase degradative pathway, the parasite instead detoxifies heme by forming crystalline FPIX within the DV, called hemozoin (Hz) or “malaria pigment”.^21–23^ Antimalarial drug therapy has long taken advantage of this physiology unique to Plasmodia, Schistosoma, and a handful of other organisms by perfecting the use of compounds such as chloroquine (CQ) and dihydroartemisinin (DHA) that, among other molecular effects, poison Hz crystallization.^24–27^ One consequence of common antimalarial drug therapy is that the parasite maintains higher-than-normal non-crystalline levels of redox-active FPIX. As emphasized earlier by Ginsburg and others,^28,29^ this FPIX biochemistry has major implications for parasite and host RBC redox homeostasis. It has long been suspected that redox biochemistry might even be perturbed in drug-sensitive versus drug-resistant malarial parasites. These observations led to studies that examined the expression of enzymes involved in GSH/GSSG cycling in drug-sensitive vs drug-resistant parasites (e.g.,^29^). Some of these studies found differences between drug-sensitive and drug-resistant parasites, but the results did not lead to a uniform explanation for drug resistance. However, to our knowledge, no direct measurements of parasite GSH and accompanying redox homeostasis have been possible. Thus, the question of whether drug-induced DV redox perturbation is linked to drug resistance phenomena, which threaten the lives of millions around the globe,^30^ remains.

One approach to measuring malarial parasite redox biochemistry is exemplified by the elegant work of Katja Becker and colleagues, who used fluorescent bioprobes and biochemical methods to measure GSH and/or redox potential for the cytosol, apicoplast, and mitochondrial compartments of the parasite within the infected RBC (iRBC).^31,32^ These redox probes are essentially green fluorescent protein (GFP)/glutaredoxin chimeras that are easily proteolyzed within the DV, so no direct measurements of the DV redox biochemistry were possible. There is a need for new methods that rely on nonproteolyzable small molecule probes.

One such new method is suggested by the work of Cho and Choi^33^ and Jiang and colleagues,^34,35^ wherein the highly fluorescent coumarin ring was derivatized to develop fluorescent GSH sensors. Such molecules are resistant to proteolysis and, in theory, could be derivatized further to localize them to intracellular compartments of the malarial parasite.

Jiang et al. begin their development of coumarin-based redox sensors using diethylamino coumarin since addition of diethylamino to various fluorophores increases their quantum yield.^36^ This led to the design and synthesis of “ThioQuant Green” (TQG), which incorporates a side chain that directs GSH covalent binding via Michael addition,^34^ making the diethyl coumarin fluorescence responsive to physiologic changes in [GSH]. In a follow-up study,^35^ the diethylamino group was replaced by an azetidine ring, as the rigidity of the azetidine was suspected to further increase the quantum yield of the fluorescent probe.^36,37^ This was indeed found to be the case; however, to develop this probe, Jiang and colleagues began with azetidine coumarin aldehyde synthesized in an undescribed fashion by an external source, which added significantly to the cost. Regardless, the GSH Michael addition side chain was further modified by adding a cyano group to accelerate the Michael addition of a thiol.^35^ An acetoxymethyl ester (AM) derivative of this molecule was synthesized to create “Real Thio [RT]”, wherein ubiquitous cytosolic esterases convert the diffusible AM derivative to a nondiffusible, trapping it within the cell cytosol. Indeed, the probe successfully measured [GSH] in HeLa cell cytosol that was imaged using confocal microscopy.^35^ Confocal microscopic imaging of trapped intracellular fluorescent probes has distinct advantages and disadvantages. Illumination of the fluorophore is by intense monochromatic laser light, which not only allows for more robust visualization of a probe but also can promote probe photobleaching. Another disadvantage is that only certain wavelengths of laser light are available, which limits quantification of the response of some probes. In contrast, customized wide field single-cell photometry (SCP) as used within can be more easily tailored at both excitation and emission wavelengths, and for reduced photobleaching, to optimize biologically relevant signals from trapped intracellular fluorophores (e.g.,^38–40^ ). We first describe how to dramatically lower the cost of probe synthesis by beginning with umbelliferone, to which azetidine is added via a palladium-catalyzed cross-coupling reaction. Our subsequent synthesis of 7-azetidinyl-3-formyl coumarin results in a savings of at least $2500/g toward synthesis of these useful probes, facilitating further functionalization and thereby allowing rapid imaging of [GSH] in live intraerythrocytic malarial parasites under constant physiologic perfusion using previously perfected probe trapping methods and customized SCP as described.

Specifically, we localize a dextran-conjugated probe synthesized from a low-cost precursor to the DV of malarial parasite strains C2^GCO3^ and C4^Dd2^ as well as strains CamWT and CamWT^K13‑580Y^. These strains are reverse-engineered genetically matched pairs of chloroquine-sensitive (CQS) vs resistant (CQR) and artemisinin-sensitive (ARTS) vs resistant (ARTR) parasites, respectively, and they have been described in detail elsewhere.^41,42^ We note that CamWT^K13‑580Y^ is created from CamWT, which is also CQR. That is, to our knowledge, all ARTR malarial parasites analyzed so far from the field or created in a laboratory are also CQR. As best we can ascertain, no viable reverse genetically engineered ARTR parasites have been created from or derived from parasites that are both CQS and ARTS. We also note that all CQR parasites have similar CQ IC_50_ shifts that indicate similar (∼10-fold) resistance to CQ growth inhibition due to PfCRT mutation but may have very different LD_50_ shifts, showing different levels of resistance to the parasiticidal effects of CQ that are manifested at higher drug doses.^43,44^ Taken together, this suggests that some (but perhaps not all) biochemical changes that accompany the emergence of the variety of CQR phenotypes found in malarial parasites may be necessary for the acquisition of ARTR status. We use probe localization within the live parasite DV to quantify [GSH]^DV^ under perfusion with physiologic perfusate either – or + pharmacologically relevant doses of CQ or dihydroartemisinin (DHA). The results are important for elucidating connections between malaria parasite redox homeostasis and resistance to CQ and DHA, which threaten the lives of millions annually. ^2^

## Materials and Methods

### Materials

P. falciparum strains were obtained from MR4 (Malaria Research and Reference Reagent Resource Center, BEI Resources) or were a kind gift from Dr. D. Fidock (Columbia University, NY, NY). Human O+ heat-inactivated serum and human O+ blood in citrate phosphate dextrose were purchased from Valley Biomedical (Winchester, VA) and BioIVT (Hicksville, NY). Custom 5% CO_2_, 5% O_2_, 90% N_2_ gas blends were purchased from Robert’s Oxygen (Rockville, MD). RPMI-1640, poly-l-lysine solution, and Giemsa stain were purchased from Sigma-Aldrich (St. Louis, MO). Albumax II lipid-rich bovine serum albumin and amino-functionalized dextran were purchased from Thermo Scientific (Waltham, MA). All other chemicals for cell culture and buffers were purchased as biochemistry grade from Fisher Scientific (Hampton, NH). All chemicals for synthesis were purchased as reagent grade or better from Fisher Scientific (Hampton, NH), Sigma-Aldrich (St. Louis, MO), Combi-Blocks Inc. (San Diego, CA), or Tokyo Chemical Industries (Tokyo, Japan). Chloroquine (CQ) and dihydroartemisinin (DHA) were purchased from Sigma-Aldrich and Tokyo Chemical Industries (Tokyo, Japan), respectively.

## Methods

### Washing Erythrocytes

Erythrocytes were washed by aliquoting 13 mL of buffered RBC and centrifuging at 2000 g for 7 min. The supernatant was removed by aspiration, and the remaining erythrocytes were resuspended in incomplete media (RPMI-1640 with 24 mM NaHCO_3_, 11 mM glucose, and 750 μM hypoxanthine, pH 7.4) to bring the total volume in the tube to 14 mL. Subsequent washing steps were performed a total of 4 times before the washed erythrocytes were used. RBCs washed in such a fashion can be used for parasite culture for up to 7 days if stored at 4 °C.

### Cell Culturing

P. falciparum strains were maintained as previously described^45^ with minor changes. Cultures were initiated from frozen glycerolyte backups at parasitemia levels higher than 5% in the ring stage, preserved in liquid N_2_. Thawing was performed by the gradual addition of sterile 12% NaCl in DI water (1 volume of NaCl solution to 5 volumes of glycerolyte backup), followed by the gradual addition of 5 mL of 1.8% NaCl in DI water and then 5 mL of 0.9% NaCl with 0.2% glucose in DI water. Cultures were maintained in RPMI-1640 supplemented with 25 mM HEPES, 24 mM NaHCO_3_, 11 mM glucose, 750 μM hypoxanthine, 20 mg/mL gentamycin sulfate, and 5 g/L albumax II. Culture flasks were then purged with 5% CO_2_, 5% O_2_, and 90% N_2_ by aeration of the cell culture suspension using a cotton-plugged Pasteur pipet. In general, cultures were kept at 2% hematocrit with parasitemia <10% and monitored by Giemsa staining.

### Synchronization

Parasite cultures were synchronized as described previously^46^ by treating them three times with a 5% D-sorbitol solution in DI water, leaving only ring-stage and early trophozoite-stage parasites each time.

### Loading Erythrocytes with Dextran Dyes

Erythrocytes were loaded with dextran-conjugated dyes through the hypotonic lysis/hypertonic sealing technique as previously described^40^ using an approach developed earlier by Krogstad and colleagues^47^ with minor modifications. Briefly, dextran dye was dissolved in DI water at a 20 mg/mL concentration, and 20 μL was added to 220 μL of a hypotonic loading solution containing 5 mM HEPES, 11 mM d-glucose, and 2 mM MgATP, at pH 7.4. To the hypotonic loading solution was added 100 μL of freshly washed erythrocytes, and the solution was mixed by continuous inversion of the tube for 10 min. The erythrocytes were then resealed by the addition of 250 μL of a hypertonic solution containing 280 mM NaCl, 40 mM KCl, 11 mM d-glucose, and 2 mM MgATP, at pH 7.4. The probe-loaded erythrocytes were then washed twice by centrifugation at 1000 g for 3 min, followed by aspiration of the supernatant and washing with 10 mL of incomplete media (RPMI-1640 with 24 mM NaHCO_3_, 11 mM glucose, and 750 μM hypoxanthine, pH 7.4). Small cultures were then started by the addition of 45 μL of the probe-loaded RBC pellet to 5 mL of complete media (either with O+ human serum or Albumax II, see *cell culturing* section in the methods) containing iRBCs at the schizont stage. Upon eventual iRBC lysis, released free merozoites then infected the probe-loaded RBCs and internalized the probe within the DV.^40^ Cultures were maintained for up to 3 cycles (6 days) and were sustained by changing media every 2 days as described (see *cell culturing* section in methods). Loading was confirmed by examining probe-loaded RBCs before and after infection using a widefield microscope in fluorescence mode (see Results).

### Spinning Disc Confocal Microscopy (SDCM)

Our customized SDCM microscope setup is as described previously^40,46^ with some modifications. In brief, the camera is an Andor iXon DV887 fitted to a Yokogawa CSU 21 confocal scanning unit and a Nikon Eclipse TE2000-U inverted microscope. The lasers were Coherent-pumped diode lasers at 405 nm (80 mW), 491 nm (100 mW), 561 nm (50 mW), and 642 nm (150 mW) (Coherent Corp., Saxonburg, PA), controlled by a laser control unit (Spectral Applied Research Inc., Richmond Hill, ON, Canada). The stage was an ASI imaging stage with a 150 μm piezoelectric Z-stage controlled by an ASI MS-2000 stage controller (Applied Scientific Instrumentation, Eugene, OR). Images were acquired through SlideBook 6, fluorescent images were deconvolved in AutoQuant X2 (Media Cybernetics Inc., Rockville, MD), and analyzed using Imaris version 7.6.3 (Oxford Instruments, Abingdon, UK).

### Single-Cell Photometry (“SCP”; Customized Widefield Fluorescence Microscopy)

To quantify probe response vs time within the live iRBC parasite, we customized a widefield microscope system based on a Nikon Diaphot epifluorescence microscope base with a Zeiss Axiocam 305 mono camera fitted to the side port (see also references 38–40 for our SCP methods). Fluorescence illumination was provided by a 175 W xenon arc lamp in a LAMBDA LS lamp housing (Sutter Instrument Company, Novato, CA). Excitation wavelengths were controlled by a LAMBDA 10-2 controller with a 10-position filter wheel (Sutter Instrument Company), fitted with ET385/20× and ET520/20× bandpass filters (Chroma Technology Corp., Bellows Falls, VT), and a blank aluminum disc for blocking light (see Results). To collect probe fluorescence at multiple wavelengths in a quasi-ratiometric format, a customized filter cube with a multi-dichroic beamsplitter and a dual-wavelength emission filter fabricated by Chroma Technology Corp. (Bellows Falls, VT) was used. Combined with dual-wavelength excitation control of arc lamp illumination, and as shown in Results, this allowed for an optical path substantially more amenable to collecting trapped intracellular fluorescence ratios relative to confocal microscopy methods that cannot be as easily controlled with regard to intensities and wavelengths of illumination. Detailed spectral characteristics of this custom filter cube are described in Results and the supplemental file (Figure S3). In brief, the cube uses a unique dichroic mirror and a combination of filters to provide an optimized optical path superior to previous confocal microscopic imaging of azetidinyl-coumarin-based probes (see below and caption Figure S3). All SCP imaging hardware was controlled by a Dell computer running ZEN blue 3.5 (Carl Zeiss AG, Jena, Germany [below, Figure S4]). Unless otherwise noted, an imaging experiment under perfusion was conducted as follows: cultured iRBCs were attached to polylysine-coated coverslips^40,46^ and immediately perfused with EBSS buffer (37 °C, equilibrated with 5% CO_2_/5% O_2_/90% N_2_ custom gas blend; Roberts Oxygen, Rockville, MD). Samples were alternately illuminated at 385 and 520 nm for 500 ms as 485 and 565 nm fluorescence emission were captured, respectively, with excitation light blocked for 100 ms and 1.9 s after the two illumination steps, to prevent photobleaching between fluorescence emission captures. Via this protocol, a multichannel image is acquired every 3 s with 1.9 s of darkness between images to allow for fluorophore recovery. Perfusion experiments were carried out essentially as described previously.^38,39,46,48^ >2 individual cultures (each culture independently loaded with probe) were used to average data from *n* individual cells (unless otherwise noted, *n* ≥ 70).

### Chemistry

#### Routine NMR Spectroscopy

Chemical synthesis intermediates were routinely tested by NMR to quantify their structure and examine their purity. ^1^H NMR spectra were acquired on a Varian 400 MHz 400-MR NMR spectrophotometer (Agilent, Santa Clara, CA). Generally, samples were prepared in either deuterated chloroform (CDCl_3_) or deuterated DMSO (DMSO-*d_6_*), both obtained from Cambridge Isotope Laboratories (Tewksbury, MA). Routine ^1^H NMR spectra were acquired at room temperature using auto shim and represent the average of between 16 and 64 scans depending on sample concentration.

#### Liquid Chromatography–Mass Spectrometry (LCMS)

For identification of chemical compounds by LCMS, we used an Agilent 1260 Infinity II LC System connected to an Agilent iQ single quadrupole mass spectrometer with electrospray ionization (Agilent, Santa Clara, CA) and an Accucore C18 HPLC Column from Thermo Fisher Scientific (Waltham, MA). The solvent systems used were water (solvent A) and methanol (solvent B), at a flow rate of 0.4 mL/min with a gradient of 5–95% solvent B over 5 min.

#### Thin-Layer Chromatography (TLC)

From a given reaction, a dilute sample of each starting material, reagent, and reaction product was spotted separately on a TLC plate about 5 mm from the bottom. Plates were placed in a glass chamber with the mobile phase (either dichloromethane (DCM) and methanol, ethyl acetate and hexanes, or acetonitrile and water) touching the bottom. Visualization of the TLC plate was done either by visually examining UV-active spots under a UV lamp (254 nm/365 nm illumination wavelengths) or by staining with permanganate (50 mM KMnO_4_, 350 mM K_2_CO_3_, 15 mM NaOH in water) and visualizing by heating with a heat gun.

#### X-ray Crystallography

Crystals were grown by adding ∼1–2 mg of the sample into a small glass vial and then dissolving it in DCM. The small vial was left uncapped and placed into a larger capped vial containing a small amount of hexanes. This allowed for the slow diffusion of hexanes into the DCM, and single crystals of the compounds described here were routinely observed 1–4 weeks later. Crystallographic X-ray data were collected on Bruker D8 Quest at 100 °K.

#### Fluorescence Spectroscopy

Fluorescence data collected in cuvettes were obtained using either a PTI QuantaMaster fluorescence spectrophotometer (HORIBA Scientific, Piscataway, NJ) or a Cary Eclipse fluorescence spectrophotometer (Agilent, Santa Clara, CA). Data were collected in 1 × 1 cm quartz cuvettes with a slit width of 2 nm for both excitation and emission wavelengths. Fluorescence titrations of probes were performed by preparing stock solutions at twice the desired final concentration. Samples were then prepared by mixing the probe solution 1:1 with the GSH solution followed by incubation in the dark at RT for 10 min. Samples were then transferred to a quartz cuvette, and fluorescence was measured.

Alternatively, fluorescence spectra were collected on a Tecan Infinite M200 plate reader (Männedorf, Switzerland) using black-walled, clear-bottom plates. Fluorescence was collected as top reads with excitation and emission bandwidths of 9 and 20 nm, respectively. Solutions were prepared as described above, and 200 μL of sample was added to each plate well for analysis.

#### SCP Thin-Layer Calibration

Thin-layer calibrations were done essentially as described previously.^38,48^ In short, standard solutions of the probe, either – or + GSH, were prepared in volumes of 100–200 μL using 0.1 M pH 5.2 propionate buffer (or phosphate buffer for pH ≥ 7.0). Ten μL of the sample solution was deposited on a microscope slide, and a #1.5 coverslip was gently placed on top. After inverting the slide, thin-layer calibration samples were immediately imaged through the coverslip exactly as individual parasites would be imaged, using the same microscope settings, optical light path, and ROI (region of interest) size, as described below for SCP of live cells.

## Results and Discussion

### Novel Synthesis of Probe AzRP1-OH (4) and Its Derivatives

We modified a previously reported reversible GSH-sensing small-molecule fluorescent probe^34,35^ to image live intraerythrocytic malarial parasites under constant perfusion with a physiologic buffer. We first required the scaffold of the probe (AzRP1-OH [**4]**; Figure 1). As described by Jiang and colleagues,^35^ probe precursors are commercially available; however, these are prohibitively expensive if additional chemical derivatization is needed. We thus developed a novel synthesis of the precursor (compound **3,** Figure 1) to produce hundreds of mg of the probe (**4)** from inexpensive reagents.

**Figure 1:**
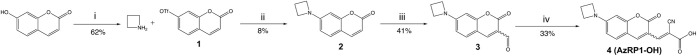
Novel synthesis scheme for an azetidinyl coumarin redox probe (AzRP1 backbone in carboxylate form, compound **4** (AzRP1-OH). Calculated synthetic yields for each step are shown beneath the arrows. Additional synthetic chemistry detail such as accompanying NMR data is reported in the SI file synthesis section (**SI 1**), but in brief, (i) (3.0 equiv) pyridine was added to 92.9 mmol umbelliferon, (1.2 equiv) trifluoromethane sulfonic anhydride was then added dropwise in DCM to yield **1**; (ii) (1.4 equiv) azetidine hydrochloride, (0.05 equiv) Pd_2_dba_3_, (0.15 equiv) 1,1’-bis­(diphenylphosphino)­ferrocene, and (5 equiv) potassium carbonate are reacted with **1** in dry 1,4-dioxane with stirring for 24 h at 80 °C; (iii) (4 equiv) phosphoryl chloride are added dropwise to DMF to activate at 55 °C and then reacted with **2** dissolved in dry DMF at 60 °C to yield 7-azetidinyl 3-formyl coumarin; (iv) (3 equiv) 2-cyanoacetic acid and (0.1 equiv) pyrrolidine in pyridine are stirred with **3** overnight to yield **4**, which we call “AzRP1-OH”).

Additional synthetic chemistry is reported in Supporting Information; in brief, 7-hydroxycoumarin is first activated by treatment with triflic anhydride to form the triflate (Figure 1). Azetidine is then added via the palladium-catalyzed Buchwald–Hartwig coupling of **1** (Figure 1) with azetidine hydrochloride. Lastly, **3** (Figure 1, right) is formed by the Vilsmeier–Haack reaction of **2** with phosphorus oxychloride (POCl_3_) in dimethylformamide (DMF). The final step, to produce fluorescent **4**, whose fluorescence is sensitive to physiologic [GSH], is performed as previously reported^35^ by reacting **3** with cyanoacetic acid to yield **4**.

As previously described,^35–37^ the addition of small, nonflexible cyclic amines increases the quantum efficiency (QE) of coumarin fluorescence compared to dimethyl-, diethyl-, and larger cyclic amines added at the same position. To explore explanations for the photophysical basis of these phenomena, we synthesized the azetidinyl C7 derivative (**2**) and compared its structure to that of the commercially available diethylamine derivative by growing single crystals of each and solving their atomic-level structures (Figure S4).

No major differences in coumarin atom electron density are found for the two molecules (Figure S4); however, the trigonal planar geometry of the attached 7-position N suggests that the bonding orbitals of this nitrogen are mostly sp^2^ like in character. Hence, the e^–^ lone pair on N is located in the p-orbital orthogonal to the plane of the coumarin moiety. This allows the lone pair to delocalize onto the coumarin ring and contribute to its fluorescence properties. While there remains some debate in the field regarding differences in QE for small cyclic amine derivatives, a prevailing idea is that of a **t**wisted **i**ntramolecular **c**harge-**t**ransfer state (**TICT** state),^49–51^ in which the amine in the excited state twists 90° so that the p-orbital is no longer conjugated with the coumarin while also fully donating an electron to the ring system during the charge-transfer step.^51^ Although our X-ray methods do not allow direct visualization of the excited state, it is conceivable that the more flexible diethylamine can nonradiatively relax to an sp^3^-like conformation, while the more rigid azetidine cannot do so as easily, resulting in less nonradiative decay, more fluorescent radiative decay,^49–51^ and enhanced QE.

Next, to target the azetidinyl probe to the DV of the parasite, the probe backbone (AzRP1-OH, **4**) was coupled to hypothesized DV-targeting groups. Several methods have been used to target probes to acidic organelles, including functionalizing them with titratable amines such as piperazines, morpholines, and quinolines, which could then trap a diffusible probe within the DV via weak base partitioning.^52^ Alternatively, for live P. falciparum, coupling the fluorophore to dextran molecules has been used to target the DV^39,40^ by using mild hypotonic “loading” of uninfected red blood cells (RBCs) followed by infection of these preloaded RBCs with live parasite merozoites^40,47^ that then ingest the dextran fluorophore and concentrate the probe within the DV. We thus synthesized both a morpholino-functionalized probe (Figure 2, **AzRP1-morph, [8]**) and several versions of dextran-conjugated probes with differing probes either without or with a 6-carbon linker to yield the **5,a,b,c** or **7a,b,c** series of compounds, respectively (Figure 2).

**Figure 2:**
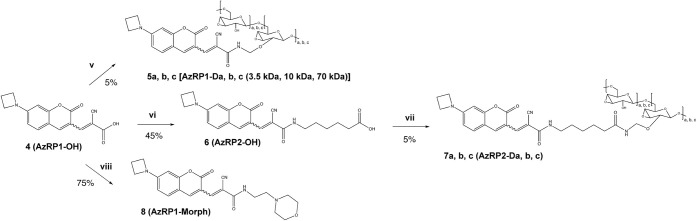
Synthesis schemes of probes derivatized from the AzRP backbone, **4** (AzRP1-OH.) The SI file synthesis section reports additional details on synthetic chemistry, but in brief, the synthesis of a dextran-functionalized probe without hydrocarbon linker, **5 a,b,c** (AzRP1-Da,b,c) (subscripts denote coupling to 3.5 kDa, 10 kDa, or 70 kDa dextran) was performed by reacting **4** (AzRP1-OH) with (0.05 equiv) amino-functional dextran, (4 equiv) 1-(3-(dimethylamino)­propyl)-3-ethyl carbodiimide hydrochloride (EDC), (4 equiv) hydroxy benzotriazole (HOBT), and (6.4 equiv) triethylamine (TEA) in dimethyl sulfoxide (DMSO). The synthesis of the probe with an added 6-carbon linker (**6**) (AzRP2-OH) in order to then derive the dextran-conjugated probe with the linker spaced between fluorophore and dextran allowed synthesis of **7 a,b,c** (AzRP2-Da,b,c): vi) (1.05 equiv) 6-aminohexanoic acid, (1.05 equiv) EDC, (1.3 equiv) HOBT, and (3 equiv) TEA were added to **4** in dimethylformamide (DMF) to form **6** (AzRP2-OH); vii) (0.05 equiv) amino-functional dextran, (4 equiv) EDC, (4 equiv) HOBT, and (6.4 equiv) TEA were reacted with **6** in DMSO. Lastly, viii), the synthesis of the morpholino-functionalized probe, **8** (AzRP1-morph) was from **4** (AzRP1-OH) reacted with (1.5 equiv) 2-morpholinoethylamine, (2.8 equiv) EDC, (2.8 equiv) HOBT, and (4.7 equiv) TEA in DMF.

The coupling reactions are in all cases performed with standard amide coupling reagents under mild conditions (Figure 2). Coupling reactions were set up to result in ∼1:1 stoichiometry (probe: dextran amino groups) for the final product; some unreacted probe was typically visible during the purification of early trial syntheses. To quantify the purified fluorophore:dextran stoichiometry, we measured the absorbance of a solution of AzRP2-OH (**6**) at 5.5 μM and solutions of purified dextran probe conjugates at 5.5 μM. Knowing both the extinction coefficient of the probe and the dextran mass, and noting that the probe mass is trivial relative to the dextran mass, we then computed the molar stoichiometry of fluorophore:dextran (e.g., 1.01 for **7c** [AzRP2-Dc] used here; c.f. Figure S1).

### Subcellular Localization and Calibration of Probes

The AzRP1-morph probe (**8,** Figure 2) was initially synthesized with the hope that it would localize to the DV by passive loading, since the DV is acidic (pH 5.2–5.6) and an added morpholino moiety should theoretically concentrate the probe within the DV through weak base-trapping^53–55^ (the p*K*_a_ of the amine in *N*-ethylmorpholine is ∼7.6–7.7,^56^ near parasite cytosolic and perfusion buffer pH). However, when iRBCs were incubated with 10 μM **8** (AzRP1-morph), surprisingly, they showed excellent parasite cytosolic concentration (Figure 3A), presumably because of a yet unknown high-affinity morpholino-probe target within the cytosol to which the probe binds.

**Figure 3:**
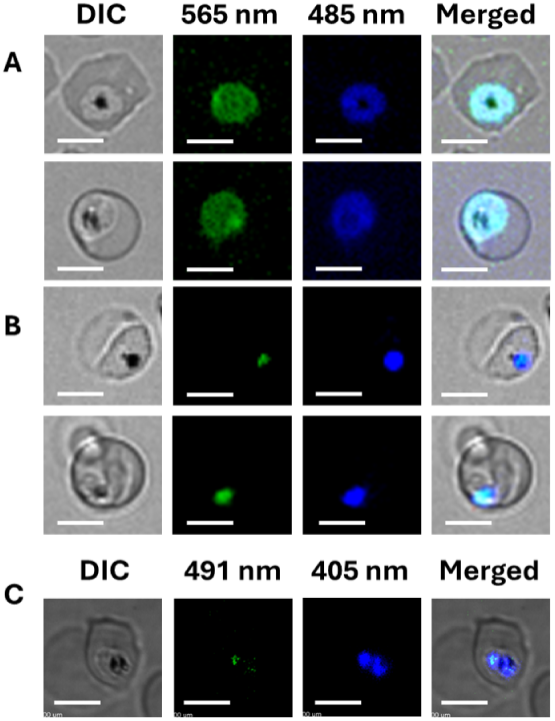
Localization of probes measured by either widefield microscopy (A, B) or SDCM (C). (A) AzRP1-morph (8) stained iRBC showing cytosolic probe localization in P. falciparum C4^Dd2^ parasites. (B) AzRP2-Dc (7c) DV staining for an iRBC after preloading RBCs and infection with C4^Dd2^ merozoites as described.^46,47^ (C) Representative SDCM image of AzRP2-Dc (7c) localized to the parasite. Optically dense Hz within the parasite DV is clearly visible by DIC (left), with AzRP2-Dc fluorescence immediately proximal to Hz in x,y, and z dimensions (C; proximity histograms not shown). Laser excitation wavelengths used for SDCM were 405 and 491 nm, which overlap with, but are not at peak, excitation of maximal fluorescence of probe:GSH conjugate and unbound probe, respectively (maximal excitation is at 385 and 520 nm, respectively, see Figure S3). Fluorescence emission was measured for each channel using 460 ± 25 nm and 565 ± 25 nm filtering. Scale bar = 5.0 μm in each panel.

Fortuitously, using the imaging methods described below, we were able to use **8** to quantify cytosolic [GSH] and found that [GSH]^cyt^ for live iRBC C4^Dd2^ parasites under perfusion is 2.7 mM ± 0.5 mM, similar to previous results found for other strains of P. falciparum (NF54 ^57,58^ and FCBR^59^) or a rodent parasite species (P. berghei ANKA-GFP).^57^ These earlier determinations were obtained upon either complete or partial lysis of iRBCs and measurement of GSH concentration using Ellman’s assay, which reported GSH concentrations in the range of 0.5–2.3 mM.^28,31,58,60–63^ However, lysis of cells exposes their interior to oxidation, which then affects such measurements (acting to lower measured cytosolic [GSH]). In contrast, *in situ* intracellular imaging of trapped probes, as done here with **8,** does not risk oxidation upon lysis and the concomitant decrease in measured [GSH]. Regardless, using the average value for [GSH]^cyt^, a GSH redox potential of ∼−300 mV^58^ for the parasite cytosol, as previously measured by Becker and colleagues with a hGrx1-roGFP2 probe, and the Nernst equation with a generally accepted *E*_0_ of GSH of −240 mV at pH 7.4, we estimate cytosolic [GSSG] to be ∼80 μM.

We performed thin-layer calibration of **7c** (AzRP2-Dc) and **8** in the complete absence of GSH using SCP identical to that used for live iRBCs (see Methods) and obtained similarly excellent quadratic fits for the relationship between [GSH-free probe] and fluorescence intensity (e.g., Figure S8A and see below, following section).

**4** (AzRP1-OH), **7a,b,c** (AzRP2-Da,b,c), and **8** (AzRP1-morph) (Figure 2) were also examined by fluorescence spectroscopy and yielded similar spectrophotometric properties relative to previously reported “RealThiol” (RT)^35^ (the RT structure is published in Figure 1 in citation^35^) including similar *K*’_d_ values for GSH (calculations not shown, see below). We found that **4**, **7c,** and **8**, as expected, all reacted readily with GSH as anticipated (see below and^35^). In contrast, **5c** (AzRP1-Dc; probe conjugated to 70 kDa dextran without a 6C linker, c.f. Figure 2) showed a poorer response upon titration vs GSH (not shown), leading us to hypothesize that steric interference in the Michael addition of GSH to the cyano group-containing side chain (Figure 4) arises when the probe moiety is closer to dextran (Figure 2), thereby compromising the GSH binding-dependent signal. However, the more flexible AzRP2-Dc (**7c**) did not suffer from this steric hindrance (Figure 4).

**Figure 4:**
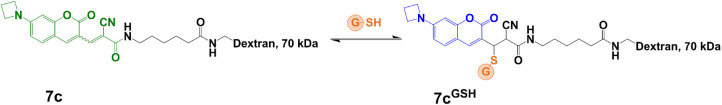
Reversible Michael addition reaction between compound **7c** (AzRP2-Dc) and glutathione (GSH). The free probe (left, green) exists as the *cis* and *trans* isomers, as indicated by the wiggled bond. The colored portions of the probe indicate fluorescent moieties and symbolize the blueshift observed upon thiol binding proximal to the cyano group.

Upon plotting the relative 485 nm (blue) and 565 nm (green) fluorescence emission intensities measured upon alternate 385 or 520 nm excitation in these experiments, the GSH *K*“_d_ for the probes could be calculated from sigmoidal curve fits and was found to be 3.9 mM, 11.4 mM, and 5.4 mM for **4**, **7c,** and **8**, respectively, in general agreement with previously reported values for this class of probe.^35^ Not surprisingly, using a 6C linker to couple **4** to dextran of 3 different masses (3.5 kDa, 10 kDa, and 70 kDa; c.f. Figure 2), we found that the 3.5 kDa dextran-coupled probe (**7a**) showed good reactivity vs GSH with a *K*”_d_ of 5.3 mM as measured by relative fluorescence (Figure 5, left), and that morpholino probe fluorescence was equally responsive to changes in [GSH], yielding a *K*’_d_ for GSH of 5.4 mM (not shown).

**Figure 5:**
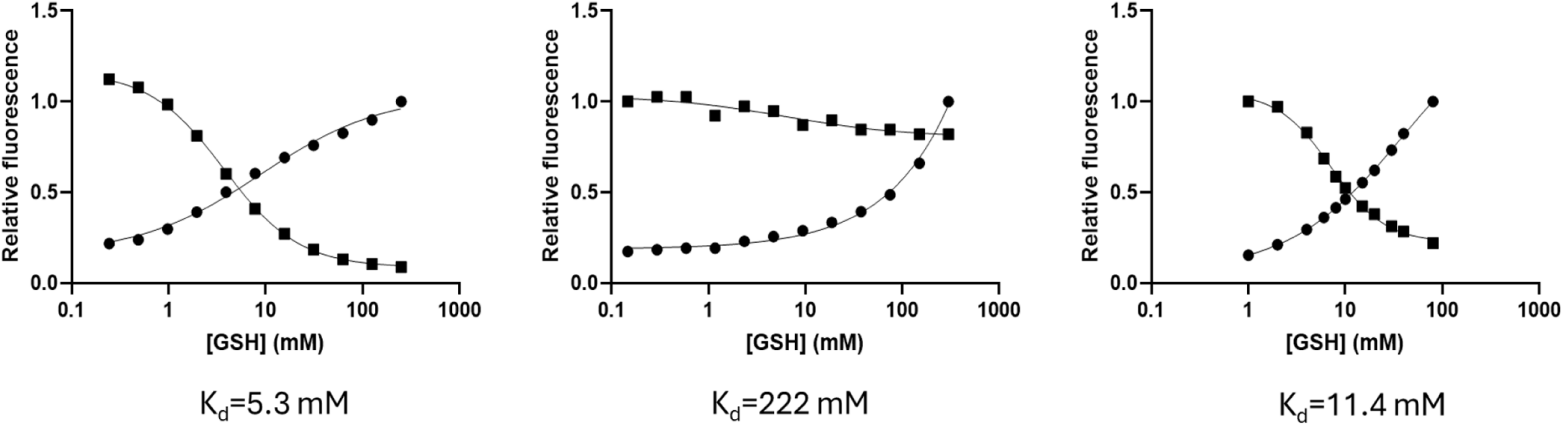
Relative fluorescence intensity for either 385 nm excitation/485 nm emission (circles) or 520 nm excitation/565 emission (squares) for 3 different dextran mass AzRP2-D probes. Left: 3.5 kDa (**7a**); middle: 10 kDa (**7b**); right: 70 kDa (**7c**) dextran. Sigmoidal curves are fitted to each function, and the dissociation constant is calculated as the intercept of the two sigmoidal curves^34,35^ and provided below the graphs.

Curiously, however, the 10 kDa dextran derivative with linker (**7b**) did not show similar reactivity relative to **7a** or **7c**, with a GSH *K*’_d_ estimated to be ∼222 mM (Figure 5 middle), similar to that for any of the three probes conjugated to dextran without a 6C linker (**5a,b,c** above). However, as previously mentioned, the 70 kDa dextran-based probe (**7c**) was found to have a useful *K*’_d_ of 11.4 mM (Figure 5 right). Fortuitously, uninfected RBCs could be efficiently loaded with **7c** but not the 3.5 kDa and 10 kDa dextran versions (**7a,b**), even though both were similarly fluorescent *in vitro*. Thus, **7c** was used in all subsequent work to image the parasite DV.

**Figure 6:**
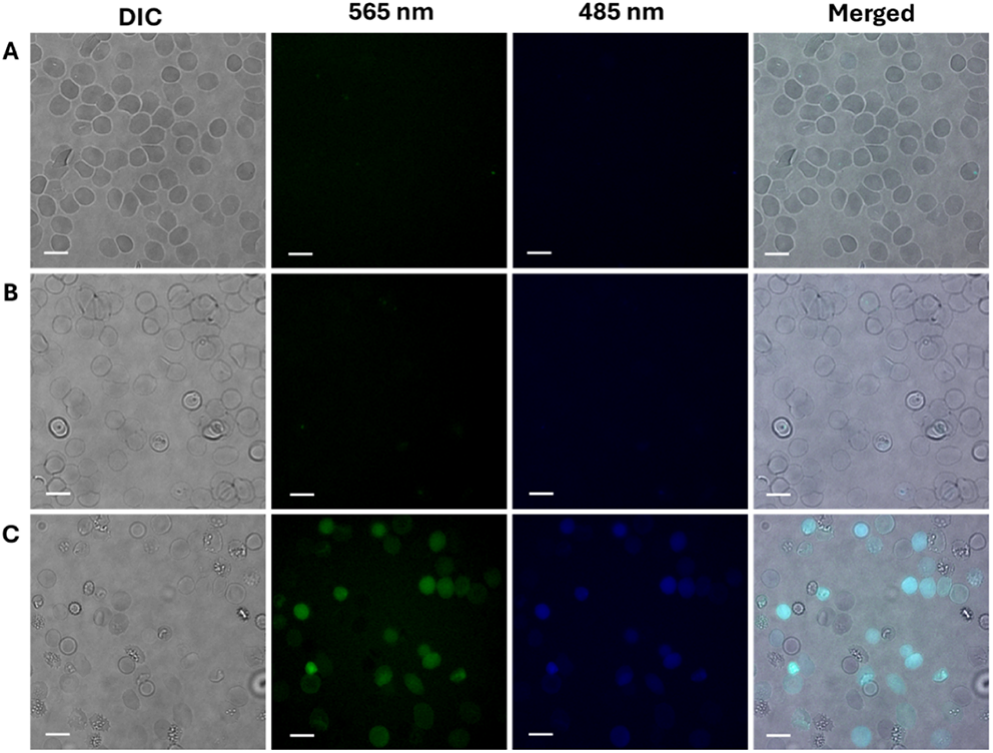
Fluorescence of RBCs hypotonically preloaded with (A) **7a** (3.5 kDa AzRP2-Da), (B) **7b** (10 kDa AzRP2-Db), and (C) **7c** (70 kDa AzRP2-Dc). Only AzRP2-Dc is effectively loaded into and retained in RBCs with our procedure to easily allow for clear visualization within parasite DV after uptake of the probe by live parasites (c.f. Figure 3 above).

### Probe Performance vs pH and in Live Parasites

To image and probe within the live intraerythrocytic parasite, we first customized both the optical path and our home-built SCP apparatus (Figures S5 and S6). Also, since the DV of P. falciparum is mildly acidic (pH ranging from 5.2 to 5.6 for CQR and CQS parasites, respectively,^38–40^ we tested whether any measurements we made could be influenced by acidic pH and found that between pH 5.0 and 7.0, there were no significant changes in the GSH-dependent response of the probe as measured by fluorescence ratios (Figure S7). Thus, a direct comparison of redox-dependent entrapped probe responses for CQS vs CQR parasite strains using SCP is immediately possible.

As found earlier for dextran-conjugated pH probes^40^ when RBCs are loaded with AzRP2-Dc, there is anticipated to be some variability in loading efficiency, and as a consequence, the resulting concentration of the probe within the parasite DV could vary slightly. To test whether this would complicate any fluorescence imaging experiments, we calibrated probe response vs [GSH] at varied concentrations of the probe in either bulk solution (Figure S8B) or in thin-layer calibration (Figure S9) using the same customized filter cube (Figure S5) and widefield microscope (Figure S6) as used for cells. We found that the relative [GSH]-dependent change in the fluorescence ratio was essentially independent over a range of 2 orders of magnitude of probe concentration (Figures S8B and S9). That is, the [GSH]-dependent quasi-ratiometric response measured by SCP using the described customized optical path (Figure S5) is nearly probe concentration-independent within a 100-fold concentration range from 200 nM to 20 μM, which spans the expected [probe]^DV^. Knowing this, we found that data sets were best fit by quadratic equations that could be averaged together to yield a convenient formula for extrapolation of [GSH]^DV^ from fluorescence ratio data regardless of precise [probe]^DV^ (Figure S9).

### Probe Imaging within Live Parasite DV

Regardless, we also measured probe concentration within individual parasite DVs for populations of parasites that were loaded with **7c** (see^40^ and Methods, Figure 3) and then fully oxidized by perfusion with 5 mM H_2_O_2_ to quantify parasite DV probe loading across a population of cultured intraerythrocytic parasites. [Probe]^DV^ was then calculated from the plateau of the 385 nm excitation/485 nm emission intensity channel after 5 min of perfusion with peroxide (Figure 7) for one cell at a time. Peroxide at this concentration was determined by titration to fully dissociate GSH from the probe within minutes (time-dependent data not shown), thus normalizing probe fluorescence intensity to be dependent upon [probe] alone. That is, 5 mM peroxide perfusion of probe-loaded live parasites that might have subtly different [GSH]^DV^ can be used to precisely quantify [probe] within individual DV by comparison to calibration of [probe] in the absence of GSH (Figure S8A).

**Figure 7:**
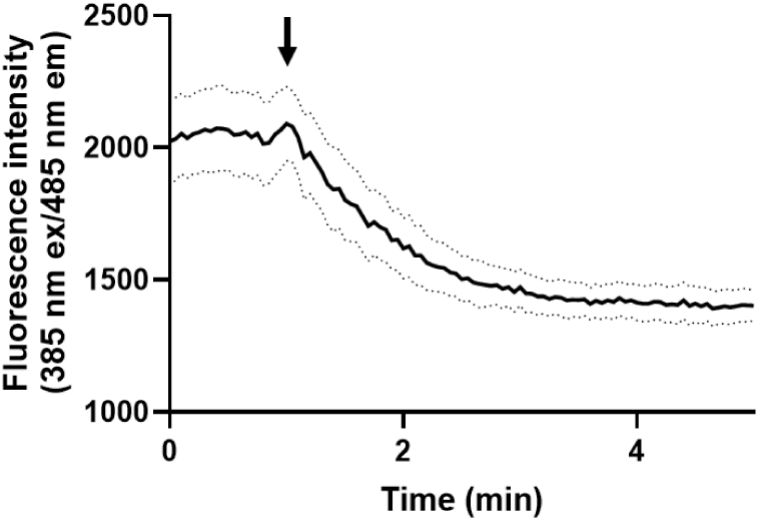
Oxidation of live parasite DV by perfusion with 5 mM H_2_O_2_. Representative single channel raw fluorescence traces record for C4^Dd2^ parasites at 485 nm emission/385 nm excitation (see Methods). Data are the average of 7 individual parasites at ∼500 nM probe; dashed lines indicate the SEM range. The arrow shows switch from EBSS buffer perfusion to 5 mM H_2_O_2_ in EBSS. A clear plateau is observed at ∼3 min, corresponding to the loss of GSH from the probe as GSH is oxidized to GSSG, concomitant with the dissociation of GSH from the cyano side chain (c.f. Figure 6) of DV entrapped AzRP2-D.

Using a quadratic fit applied to the calibration data (Figure S8A) to calculate [probe]^DV^ for individual parasites, we obtained a narrow distribution of [**7c**]^DV^ for individual live iRBC parasites within populations of the different strains (Figure 8). We find a range of **7c** DV concentrations that essentially falls between 200 and 500 nM for all examined strains and follows a narrow, normal Gaussian distribution for the populations (Figure 8, Table 1). Importantly, we also find that the distributions are similar for all strains examined in this study (Figures 8 A–D).

**Figure 8:**
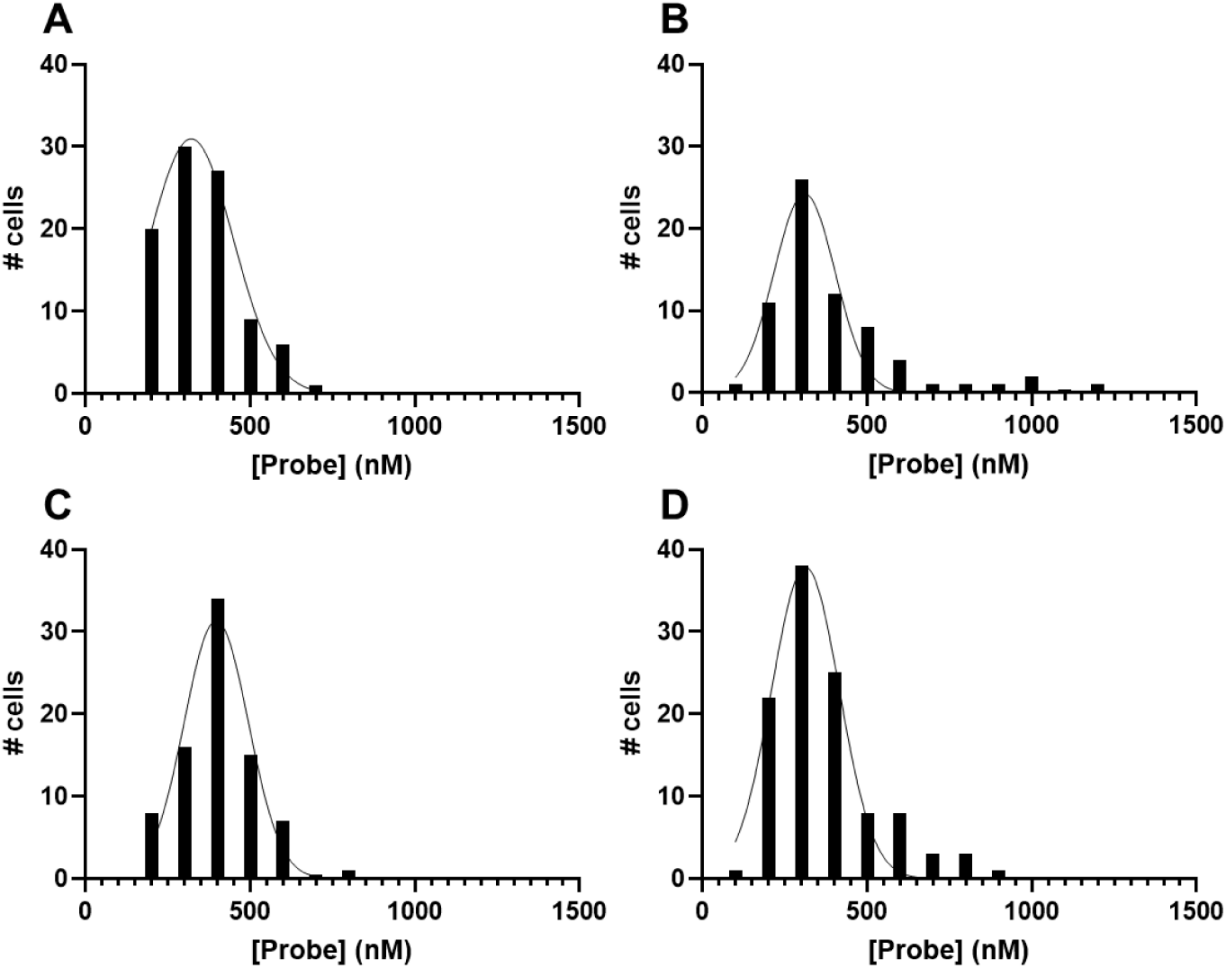
Histograms showing the distribution of [probe]^DV^ found for the 4 different P. falciparum strains examined in this work (>70 individual cells, each strain). The CQS, ARTS reverse engineered strain C2^GC03^ (**A,** top left) and its CQR, ARTS partner strain C4^Dd2^ **(B,** top right) are created by transfecting *pfcrt* constructs into a GCO3 strain that is the progeny of the strain HB3 × Dd2 genetic cross, with these constructs differing only by the *pfcrt* gene they express, either a gene encoding the GC03 (CQS) or Dd2 (CQR) PfCRT isoform.^41^ The CQR, ARTS strain CamWT (**C**, bottom left) and CQR, ARTR strain CamWT C580Y (**D,** bottom right) are a similar pair of genetically matched transfectants that differ only at a single codon in the *pfk13* gene, which leads to a C580Y substitution in the encoded PfK13 protein^42^ expressed in CamWT^K13‑580Y^. However, in this case, the parental strain (CamWT) was established from a CQR field isolate that is much more resistant to parasite death induced by CQ.^43^ Frequency distributions shown are with a binning width of 100 nM and are fitted to a Gaussian curve that overlays each histogram (thin solid lines). Average peak values ± SD for these Gaussian fits are summarized in Table 1.

**Table 1:** Average **7c** Probe Concentration in Parasite DVs as Measured by Widefield Microscopy^a^

Strain	Phenotype	[Probe] (nM)	SD (nM)	*N*
C2^GC03^	CQS, ARTS	328.2	114.4	93
C4^Dd2^	CQR, ARTS	309.9	92.8	72
CamWT	CQR, ARTS	396.9	97.4	81
CamWT C580Y	CQR, ARTR	312.4	102.9	109

a Probe concentration is the mean from the reported Gaussian distributions (Figure 8) ± SD.

These data yield average [probe]^DV^ values for the strains as listed in Table 1. A similarly narrow Gaussian distribution of the AzRP1-morph probe (**8**) is observed for parasite populations after passive loading of the cytosol (not shown, c.f. Figure 3A). From calibration data vs varied [GSH], we find an excellent ratiometric response for (**7c**) (Figures S8B and S9). That is, thin-layer calibration shows that the ratio of 485 nm emission to 565 nm emission upon alternating between 385 nm and 520 nm excitation is nearly independent of probe concentration. Analyzed another way, plotting Y-intercept data as ratio vs [probe] yields an essentially flat line with a linear slope of 0.00006 mM^1^, which is very close to zero (c.f. Figure S6B). That is, even without an obvious “isosbestic point”, these coumarin derivative probes can be used in a ratiometric fashion if imaged by SCP with a customized optical path, as we have designed (Figure S5).

Therefore, we next calculated the GSH concentration within live iRBC parasite DVs, one cell at a time, for different parasite strains ratiometrically imaged under continuous physiologic perfusion (averages are shown in Table 2; ≥ 72 individual cells for each strain). We used ratiometric **7c** signals from each live iRBC parasite DV to plot distributions of the calculated [GSH]^DV^ for individual cells (Figure S11), and although the shapes of these distributions differ slightly, we also find that under normal perfusion conditions, strains C2^GC03^ and C4^Dd2^ have similar average [GSH]^DV^ values of 11.3 and 11.6 mM, respectively (Table 2).

**Table 2:** GSH Concentration in Parasite DVs as Measured by Widefield Microscopy, Extrapolated from Calibration (Figure S9)­^a^

Strain	Phenotype	[GSH] (mM)	SEM (mM)	*N*
C2^GC03^	CQS, ARTS	11.3	0.50	93
C4^Dd2^	CQR, ARTS	11.6	0.57	72
CamWT	CQR, ARTS	7.2	0.53	81
CamWT C580Y	CQR, ARTR	10.3	0.45	109

a GSH concentration is the mean ± SEM.

These strains are either CQS (C2) or CQR (C4) solely by virtue of *pfcrt* transfection and expression of CQS vs CQR-associated isoforms of PfCRT^41^ (there is no selection with CQ used to construct these). The data thus imply that the Dd2 PfCRT isoform expressed in many CQR parasites does not, in and of itself, perturb DV redox homeostasis. In contrast, CamWT and CamWT^K13‑C580Y^ have different average [GSH]^DV^ values of 7.2 mM and 10.3 mM, respectively (Table 2).

Both Cam-derived strains are CQR, but CamWT is a field-isolated CQR strain, not analogous to the reverse-engineered C4^Dd2^ strain created in the laboratory. It is possible that additional mutations needed for higher parasiticidal resistance to CQ (resistance to the parasite-killing effects of CQ) found in CamWT relative to C4^Dd2^ perturb DV redox homeostasis).^43,44^ Additional work with more parasite strains is needed to further test this. In any case, CamWT vs CamWT^K13‑C580Y^ differ only by a single nucleotide polymorphism (SNP) in the *pfk13* gene, which leads to a C580Y substitution in the encoded PfK13 protein for CamWT^K13‑C580Y^ and a concomitant decrease in ART sensitivity as measured by the ring-stage survival assay.^42^ Mutation of PfK13 or changes in PfK13 protein abundance have previously been associated with reduced Hb catabolism^64^ as well as altered free FPIX concentrations^9^ and FPIX-DHA adduct formation.^10^ Simplistically, a higher concentration of GSH for the ARTR strain relative to its genetically matched ARTS partner strain might lead to a higher level of Fe­(II)­PPIX, which is necessary for activation of the DHA prodrug,^7,8,65^ and hence might be expected to be associated with increased sensitivity to activated DHA, not decreased sensitivity . However, the higher concentration of GSH also better detoxifies free radicals formed under drug treatment, as described below. Thus, we find that [GSH]^DV^ varies across strains of the parasite and is not influenced by the expression of CQR-conferring mutant PfCRT in and of itself but may be related to ART-based drug sensitivity as well as parasiticidal CQ resistance^43,44^ (e.g., C4^Dd2^ vs CamWT; Table 2). Analysis of additional strains will test the putative relationships we find here.

Differences between C2^GC03^ and C4^Dd2^ vs CamWT and CamWT^K13‑C580Y^ likely result from differences in their genetic backgrounds as well as their CQ parasiticidal resistance status due to different CQ selection, so analysis of the specific effects of mutant *pfcrt* or *pfK13* expression on [GSH]^DV^ can only be made reliably within pairs of genetically matched strains but not across all four strains. Of note, however, is that the GSH concentrations found in all four of these strain DVs are higher than those previously found in whole parasite lysates (which are dominated by cytosol concentrations, since cytosol volume is by far the largest of all cell compartmental volumes) or those we measured here with the similar AzRP1-morph probe (**8**) localized to the cytosol. As mentioned, lysate or cytosol [GSH] has been found to be in the range of 0.5–2.7 mM. Thus, our measurements using the same GSH-dependent fluorophore trapped in either the DV **7c** or the cytosol **8** are consistent with GSH being concentrated at least 3–4 fold within the DV, relative to parasite cytosol.

### DV Oxidative Burst under Drug Perfusion

It has been suggested that parasites may experience an oxidative burst when treated with either CQ or ART;^58–67^ however, this has only been previously examined for the parasite cytosol^32,58,67^ or whole parasite lysate.^13,66^ Since the DV is believed to be the principal site of action for CQ and ART-based drugs such as DHA and is also where the release of redox-active heme during Hb digestion occurs, we examined whether an oxidative burst was observed within the DV of live iRBC parasites upon perfusion with parasiticidal doses of either CQ or DHA. We found that consistently, regardless of strain phenotype or drug type, a fast oxidative burst was observed inside the DV within minutes of perfusion with either drug (Figure 9). This differs from oxidation seen in the cytosol of parasites treated with CQ or DHA, where a difference in redox homeostasis is only observed after incubation with the drug for ∼24 h.^58,67^ Importantly, control experiments showed that these drugs (including activated DHA vs inactivated DHA prodrug) had no significant independent effect on probe fluorescence (Figure S11).

**Figure 9:**
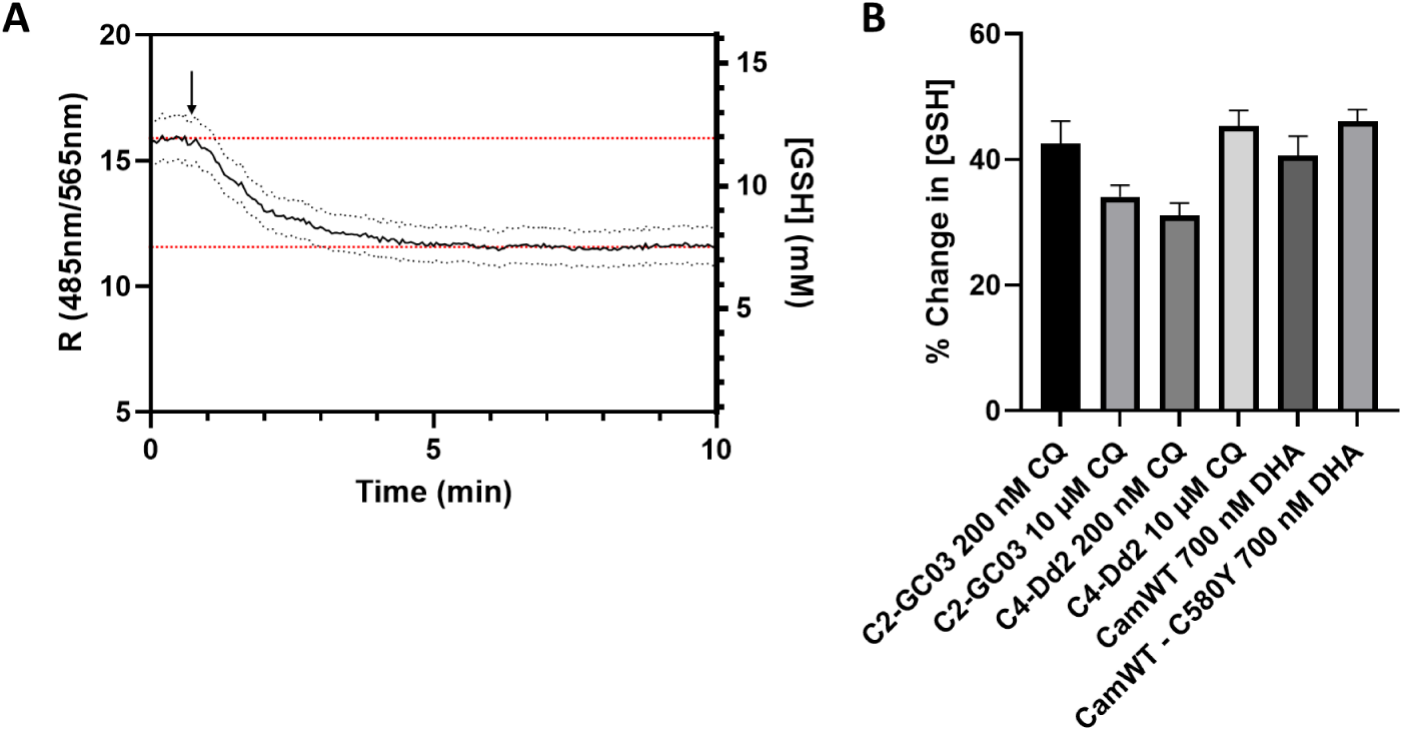
**(A)** DV GSH upon drug treatment. C4^Dd2^ **7c** response upon perfusion with LD_50_ concentration of CQ (10 μM). Cells are initially perfused with EBSS for 1 min as described (see methods); then, the buffer is changed to EBSS with drug indicated by the arrow. Trace is the average for 41 individual cells imaged separately; dashed black lines indicate ± SEM, and red dashed lines indicate plateau before (upper line) and after (lower line) treatment with drug. (**B)** Decrease in GSH quantified as a percentage of the starting [GSH] for different strains exposed to the indicated drug pulse under live-cell perfusion. Data shown are the average of ≥35 individual cells across at least 3 separate experiments, ±SEM.

In the CQS/ARTS strain C2^GC03^, we found that treatment with a concentration of CQ similar to that found in the serum of patients undergoing treatment with CQ leads to a drop in GSH concentration of ∼34%. In contrast, a 43% decrease in GSH concentration is observed upon perfusion at a lower concentration of the drug corresponding to the CQS strain LD_50_ (200 nM) (Figure 9B). It seems paradoxical that a smaller oxidative burst is observed under perfusion with a higher concentration of the drug; however, this might be explained by DV pH alkalinization caused by the higher concentration of the drug.^47^ Interestingly, when C2^GC03^ and C4^Dd2^ were treated at similar absolute [CQ], DV redox homeostasis was less perturbed for the CQR C4 strain (Figure 9B), but when they were treated with similar pharmacologically equivalent LD_50_ concentrations of CQ (C2^GC03^: 200 nM; C4^Dd2^: ∼10 μM,^43^ we found that they had similar decreases in GSH concentrations of 43% and 45%, respectively. These perturbations suggest that redox homeostasis is likely to be part of the parasite’s defense against oxidative damage within the DV caused by quinoline-based drugs such as CQ. For CamWT (CQR, ARTS) vs CamWT^K13‑C580Y^ (CQR, ARTR), the small differences in the decrease of GSH that occur upon perfusion with similar [DHA] in each strain may not be statistically significant. Upon perfusion with 700 nM DHA (a concentration similar to patient plasma levels of the drug, as used in the RSA assay to assess parasite sensitivity to DHA,^4,42^ a 41% vs 46% decrease was observed within 5 min for CamWT and CamWT^K13‑C580Y^, respectively (Figure 9B). These decreases are similar to those observed for C2^GC03^ and C4^Dd2^ when they were treated with pharmacologically matched LD_50_ concentrations of CQ (Figure 9B). Thus, we conclude that DV redox homeostasis is also relevant to the parasite response to ART-based endoperoxide drugs that also bind to free heme, but the *pfk13* mutation associated with ARTR does not immediately perturb changes in DV redox homeostasis caused by drug exposure in and of itself. Overall, we find significant changes in [GSH] upon either drug treatment, presumably as a response to the long-hypothesized oxidative damage exerted in the parasite DV by either quinoline- or endoperoxide-based antimalarial drugs upon inhibition of Hz formation from free FPIX. Redox-active ferriprotoporphyrin IX (FPIX) heme released during parasite Hb catabolism within the DV undergoes a number of pH- and redox environment-dependent reactions as it is detoxified to the fascinating pigment Hz (Figure 10).

**Figure 10:**
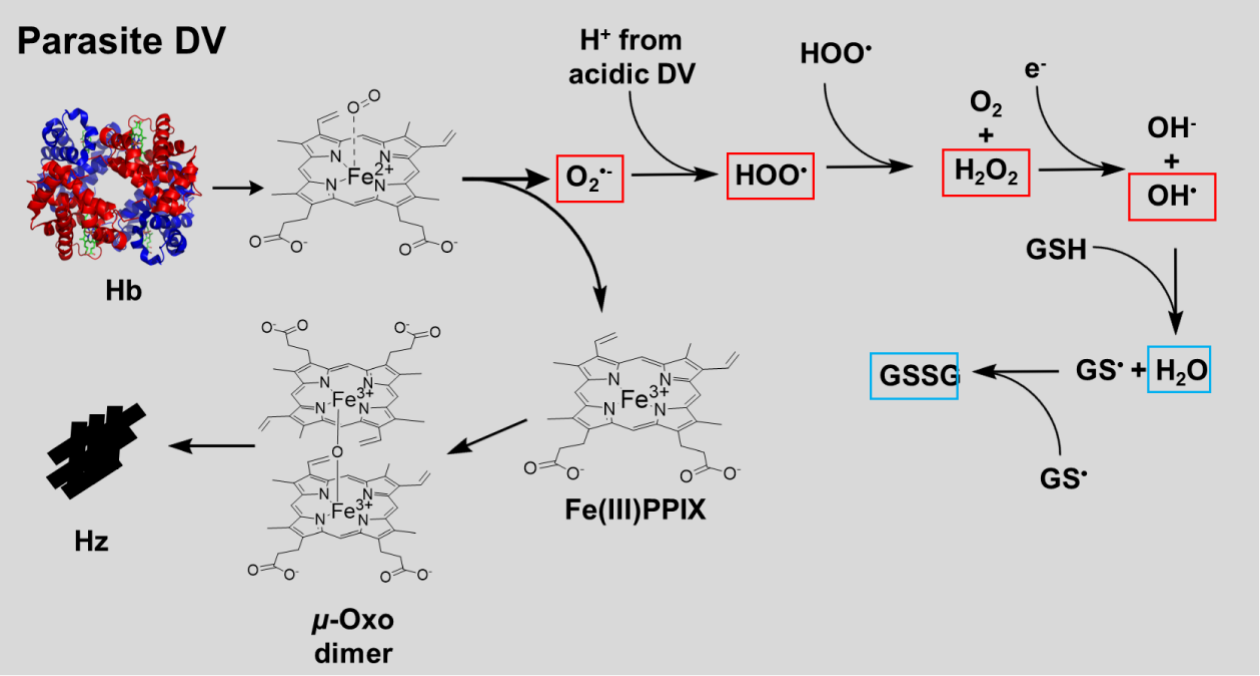
Consequences of Hb catabolism within the parasite DV. When Hb is catabolized, some oxygen bound to heme is released from the Fe^2+^/Fe^3+^ resonance state^68^ as superoxide, along with resultant Fe­(III)­PPIX. Alternatively some deoxygenated FPIX within Hb is released directly as Fe­(III)­PPIX. Both pools of Fe­(III)­PPIX progress through the μ-oxo dimer form to crystallize to Hz in order to detoxify the FPIX,^15^ while superoxide progresses through a series of reactions that form other ROS which are detoxified by GSH (see text). ROS are boxed in red, and the nonreactive detoxified products are boxed in blue. Fe­(III)­PPIX can also cycle back to Fe­(II)­PPIX via reduction by GSH. DV: digestive vacuole; Hb: hemoglobin; PPM: parasite plasma membrane; PV: parasitophorous vacuole; PVM: parasitophorous vacuolar membrane; Hz: hemozoin; RBC: red blood cell.

Quantifying these reactions is important for many reasons, among them to test whether they are related to drug exposure and drug resistance phenomena. GSH can both manipulate Fe^2+^ PPIX/Fe^3+^ PPIX ratios and scavenge HOO^•^ and OH^•^ radicals (Figure 10). Fe^3+^ PPIX predominantly forms μ-oxo dimers in aqueous solution, which are easily and quite sharply precipitated at low pH.^69^ Tethered head-to-tail dimers form from these μ-oxo dimer acid aggregates, and they then constitute the unit cell of crystalline Hz,^21^ how crystalline Hz is formed from acid-aggregated μ-oxo dimers is still not fully understood. It is likely that a balance between pH and GSH perturbations controls the overall rate of Hz formation in drug-resistant malaria. CQ and DHA bind to monomeric, dimeric, and crystalline forms of FPIX,^25–27,70^ perturbing FPIX-to-Hz conversion in multiple ways that can collectively promote DV oxidation.

To summarize, in this study, we report the following:

1) A convenient and much lower-cost synthesis of redox-sensitive AzRP1-OH (**4**) facilitates further chemical derivatization of the azetidinyl-coumarin GSH probe for use in live malaria parasites and potentially in other systems as well.

2) The differences in electron density do not easily explain variations in fluorescence quantum efficiency (QE) between linear diethylamino and cyclic azetidinyl coumarin-based probes.

3) AzRP1-morph (**8**) does not localize to the parasite DV as originally predicted, but using customized SCP instead, it reports similar parasite [GSH]^cyt^ relative to previous findings.

4) A 70 kDa dextran-coupled probe (**7c**) can be localized to the parasite DV. This is useful for characterizing drug-dependent DV redox biochemistry in drug-sensitive vs drug-resistant malarial parasites.

5) An optimized optical path for probe signal detection from live cells using SCP and a customized dichroic mirror/filter assembly cube, rather than confocal microscopy, allows for quasi-ratiometric quantification of [GSH]-dependent fluorescence signals from individual live iRBC malarial parasites under continuous physiologic perfusion.

6) Mutations in *pfcrt* do not, in and of themselves, lead to significant changes in equilibrium [GSH]^DV^.

7) Mutation of *pfk13* may be associated with small changes in resting [GSH]^DV^, but measurements with more parasite strains are needed to test this.

8) [GSH]^DV^ is higher than [GSH]^cyt^ measured for malaria parasites in this paper and elsewhere.

9) Exposure to either quinoline- or ART-based antimalarial drugs under live-cell perfusion conditions leads to significant perturbation of DV redox homeostasis for both drug-sensitive and drug-resistant parasites.

Since Fenton chemistry-based Fe­(II)­PPIX activation of the endoperoxide group within ART-based drugs has been well established,^7,8,65^ it might be expected that at lower concentrations of GSH, [Fe­(II)­PPIX] would be lower, and consequently, the rate of ART activation would also be lower, as has indeed been surmised for ARTR parasites.^10^ It then seems counterintuitive that we do not find lower [GSH]^DV^ for ARTR vs ARTS transfectant strains, and in fact, it appears that the ARTR CamWT^K13‑C580Y^ strain has a slightly higher concentration of [GSH]^DV^ vs the genetically matched ARTS CamWT strain.

However, as mentioned, GSH not only reduces Fe^3+^PPIX to Fe^2+^PPIX but also scavenges reactive oxygen species (ROS) in the parasite, such as highly reactive hydroxyl radicals (OH^•^) and hydroperoxyl radicals (HOO^•^ [Figure 10]). These are produced due to Hb catabolism, are likely the most abundant ROS within the DV, and cause a variety of cytotoxic effects. Thus, there is a balance between minimizing free [Fe­(II)­PPIX] by lowering [GSH]^DV^ vs better scavenging ROS by increasing [GSH]^DV^ that must be optimized for ARTR vs ARTS parasites. Our results suggest that the latter is more important to the survival of ARTR parasites derived from the CamWT background when they are exposed to DHA. We conclude that it is more important for ARTR parasites from this genetic background to more effectively scavenge DV ROS than to reduce the rate of conversion of the DHA prodrug.

### The Oxidative Burden Experienced by P. falciparum during Hb Catabolism in the Acidic DV

During Hb catabolism in the DV, heme could be released either as Fe^3+^ heme not bound to O_2_ or as the oxygen-bound species, where iron is in the Fe^2+^/Fe^3+^ resonant state and oxygen is concomitantly released as superoxide (O_2_^•–^).^71^ Within Hb, the heme-iron–oxygen complex is actually resonant^68^ where both iron and oxygen exist in mixed Fe^2+^/Fe^3+^ and O_2_/O_2_^•–^ states (see Figure 11). The ability of heme in Hb to bind oxygen is, of course, quite pH-dependent, with a lower level of oxygenation at lower pH^72,73^ (see below). Since CQR parasites have lower DV pH^38–40^ relative to CQS, this suggests that CQR DV experiences reduced O_2_^•–^ release during Hb catabolism and higher Fe­(III)­PPIX release vs CQS.

**Figure 11:**
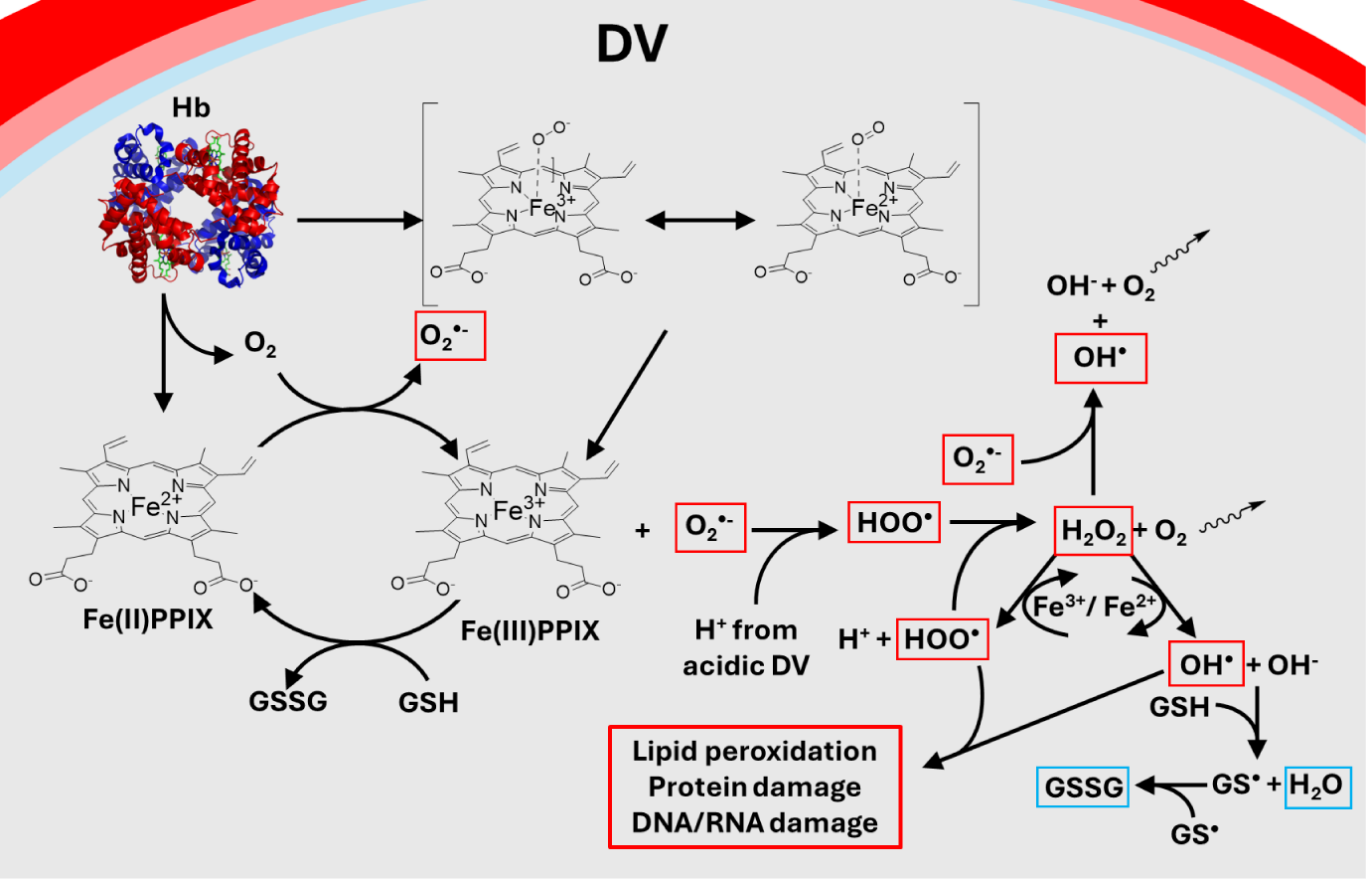
Schematic of the oxidative burden experienced within the parasite DV. When Hb is catabolized, oxygen-bound heme is initially released in a Fe^2+^/Fe^3+^ resonant state, immediately releasing superoxide and resulting Fe (III)­PPIX. FPIX progresses through the μ-oxo dimer to form Hz to detoxify FPIX (Figure 10), while superoxide progresses through a series of reactions that form other ROS that can be detoxified by GSH (see text). ROS are boxed in red, and the nonreactive detoxified products are boxed in blue.

While many eukaryotes detoxify superoxide via superoxide dismutase (SOD), and P. falciparum has been shown to import host SOD,^74^ no parasite SOD is known to be present in the parasite DV. Superoxide also undergoes spontaneous dismutation under acidic conditions, such as those found in the DV, to first form HOO^•^, followed by H_2_O_2_ and O_2_ ^75^ (Figure 11). The resulting H_2_O_2_ in turn is again redox-active and can produce HOO^•^ and OH^•^ through reactions with both Fe^2+^ and Fe^3+^ via the Fenton chemistry cycle.^76^ Both radicals cause considerable oxidative damage in a number of ways, such as lipid peroxidation, protein cross-linkage, or DNA breakage;^71^ however, two GSH molecules can detoxify these radicals to water while concomitantly converting to GSSG.^77^ Taken together, this suggests that the acquisition of an ARTR phenotype may be associated with higher radical detoxification through higher [GSH]^DV^.

The Fenton reaction promotes cycling between heme Fe^2+^/Fe^3+^ and the formation of HOO^•^ and HO^•^ radicals which GSH can then scavenge, as more precisely shown below via eqs 1 and 2. HOO^•^ can be recycled via autodismutase to reform hydrogen peroxide. These reactions work as electron sinks via formation and subsequent removal of molecular oxygen (O_2_) upon its diffusion (wiggled arrows, Figure 11).

1 $$Fe^{2+}+H_{2}O_{2}\to Fe^{3+}+OH^{-}+OH^{•}$$

2 $$Fe^{3+}+H_{2}O_{2}\to Fe^{2+}+H^{+}+HOO^{•}$$

However, a key caveat for our conclusions is the Bohr effect, which simplistically is reduced oxygenation of Hb FPIX heme promoted by lower pH. The Haldane effect can be described as the influence of CO_2_ on Hb oxygenation, but dissolved [CO_2_] of course affects pH. Bohr and Haldane thus essentially studied both pH- and CO_2_-induced shifts in the well-known Hill plot that describes Hb oxygenation vs the partial pressure of O_2_. These shifts are consequently termed the “Bohr– Haldane” effect. In any case, the parasite DV shows pH 5.6 vs 5.2 for CQS vs CQR parasites.^38–40^ How these pH levels then affect the relative release of oxygen (as superoxide) within the DV has not been measured to our knowledge but obviously could impact our measurement of drug-induced oxidative burst. We can find many investigations of Hb O_2_ vs pH for the range expected for human blood in various diseases and metabolic disorders, but to our knowledge, no data have ever been collected at pH < 6.9. On the other hand, many investigators have modeled Bohr–Haldane effects and, in the process, have written potentially useful computational algorithms. These are elegantly reviewed by Dash and Bassingthwaighte,^78^ who also provide their own algorithm to estimate the effects. Partial pressure of O_2_ and CO_2_ of course varies from the lung to distal tissues, but other than poor growth of parasites at quite low [CO_2_], we are unaware of any bias or preference for Hb import and digestion within the DV for live parasites that occur within the ranges of these gases expected in tissues. Using the average partial pressures for both gases and otherwise standard physiologic temperature, a constant concentration of^2,3^ DPG (bisphosphoglycerate, which also impacts Hb O_2_ levels^78^), and the downloaded JSim code,^78^ we expect <10% Hb to be oxygenated at pH 5.6 within the DV (vs 100% for RBC circulating within the well-oxygenated lung at physiologic pH), vs <5% at pH 5.2. This modeling suggests up to 50% less superoxide is released directly by Hb catabolism within the DV of CQR parasites relative to CQS. Precisely how much Hb is oxygenated within CQS vs CQR DV is unknown, as we are currently unable to verify the above calculations with direct experimental measurements. Regardless, for any parasite, substantial FPIX must be released from catabolized Hb as deoxygenated Fe^3+^PPIX within the acidic DV, yielding the generation of radicals as shown in Figure 11. Importantly, in the blood of a respiring human, an essentially infinite amount of dissolved O_2_ is continuously available for instantaneous diffusion into the DV, implying that with even small levels of Fe^2+^PPIX released, Hb catabolism is continuously generating superoxide as Fe^3+^PPIX is reduced back to Fe^2+^ by GSH, indicating an ironic continuous requirement for GSH to then scavenge hydroxyl radicals. Inspecting these caveats in detail warrants additional future work.

## Conclusions

We have synthesized, characterized, and demonstrated the initial application of a malaria parasite DV-specific GSH-sensing fluorescent probe. We found a similar distribution of probe concentrations within iRBC parasite DVs when loading uninfected RBCs consistently using the hypotonic loading technique and then infecting them with live merozoite culture, regardless of different parasite phenotypes in the studied populations. Using customized SCP, we quantified cytosolic and DV [GSH] for live malarial parasites, as well as the oxidative burst that accompanies drug exposure for live, genetically matched CQS vs CQR and ARTS vs ARTR reverse-engineered parasite clones.

## Supplementary Material



## Notes

1) Probe synthesis steps (Schemes 1−8) and NMR spectra (Figures S2.1−2.9); 2) Figure S3 Absorbance of Compound **6** (AzRP2-OH) vs Compound **7c** (AzRP2-Dc); 3) Figure S4 X-ray crystal structures of **CAS 54711-39-6** and Compound **3**; 4) Figure S5 Optimized optical path; characteristics of a customized filter cube used in widefield microscopy (SCP) experiments vs cube characteristics used previously by others; 5) Figure S6 SCP schematic; 6) Figure S7 Effect of pH on Compound **7c** (AzRP2-Dc) GSH – dependent quasi fluorescence 7) Figure S8 A,B Calibration of **7c** probe fluorescence vs concentration and vs [GSH]; 8) Figure S9 Extended data sets for **7c** fluorescence vs [GSH] and quadratic fits; 9) Figure S10 Fluorescence spectra of Compound **6** (AzRP2-OH) +/- GSH, +/- H_2_O_2_; 10) Figure S11 Distribution of [GSH]^DV^ found for the 4 different *P. falciparum* strains; 11) Figure S12 Effects of drugs alone on Compound **7c** (AzRP2-Dc) fluorescence; 12) Table S1−13 crystallographic data for **CAS 54711-39-6** and Compound **3** (PDF)

## Abbreviations

ACT: ART combination therapy
ART: artemisinin
ARTR: artemisinin resistance/resistant
ARTS: artemisinin-sensitive
CQ: chloroquine
CQR: chloroquine resistance/resistant
CQS: chloroquine-sensitive
DCM: dichloromethane
DHA: dihydroartemisinin
DMF: dimethylformamide
DMSO: dimethyl sulfoxide
DV: digestive vacuole
EBSS: Earle’s balanced salt solution
FPIX;: ferriprotoporphyrin IX
GSH;: reduced glutathione
GSSG: (oxidized) glutathione disulfide
Hb: hemoglobin
Hz: hemozoin
LCMS: liquid chromatography–mass spectrometry
iRBCs: infected red blood cells
PfCRT: Plasmodium falciparum chloroquine resistance transporter
PfK13: Plasmodium falciparum kelch 13 protein
POCl_3_: phosphorus oxychloride
PPM: parasite plasma membrane
PPQ: piperaquine
PV: parasitophorous vacuole
PVM: parasitophorous vacuolar membrane
QE: quantum efficiency
RBCs: red blood cells
ROS: reactive oxygen species
RPMI: Roswell Park Memorial Institute medium
RT: RealThiol
SCP: single-cell photometry
SDCM: spinning disc confocal microscopy
SNP: single nucleotide polymorphism
SOD: superoxide dismutase
TLC: thin-layer chromatography
TQG: ThioQuant Green
