# Supplementary material for: Redox Homeostasis within the Drug-Resistant Malarial Parasite Digestive Vacuole
Source: Biochemistry. 2025 May 1;64(10):2247–61. doi: 10.1021/acs.biochem.4c00750 (PMC12096432; doi:10.1021/acs.biochem.4c00750)

SUPPORTING INFORMATION

REDOX HOMEOSTASIS WITHIN THE DRUG RESISTANT MALARIAL PARASITE DIGESTIVE VACUOLE

Andreas Willems, Therese Oertel & Paul D. Roepe*

Depts. of Chemistry and of Biochemistry and Cellular and Molecular Biology,

Georgetown University

37th and O Streets NW

Washington DC 20057, U.S.A.

* email address for corresponding author: roepep@georgetown.edu

SUPPLORTING INFORMATION:

1) Probe synthesis steps (Schemes 1-8) and NMR spectra (Figures S2.1-2.9)

2) Figure S3 Absorbance of Compound **6** (AzRP2-OH) vs Compound **7c** (AzRP2-Dc)

3) Figure S4 X-ray crystal structures of **CAS 54711-39-6** and Compound **3**

4) Figure S5 Optimized optical path; characteristics of a customized filter cube used in widefield microscopy (SCP) experiments vs cube characteristics used previously by others

5) Figure S6 SCP schematic

6) Figure S7 Effect of pH on Compound **7c** (AzRP2-Dc) GSH – dependent quasi fluorescence

7) Figure S8 A,B Calibration of **7c** probe fluorescence vs concentration and vs [GSH]

8) Figure S9 Extended data sets for **7c** fluorescence vs [GSH] and quadratic fits

9) Figure S10 Fluorescence spectra of Compound **6** (AzRP2-OH) +/- GSH, +/- H_2O_2. 10)

10) Figure S11 Distribution of $[GSH]^{DV}$ found for the 4 different *P. falciparum* strains

11) Figure S12 Effects of drugs alone on Compound **7c** (AzRP2-Dc) fluorescence

12) Table S1-13 crystallographic data for **CAS 54711-39-6** and Compound **3**

1) Probe Synthesis steps and NMR spectra

Scheme 1, Compound 1 – 2-oxo-2H-chromen-7-yl trifluoromethanesulfonate

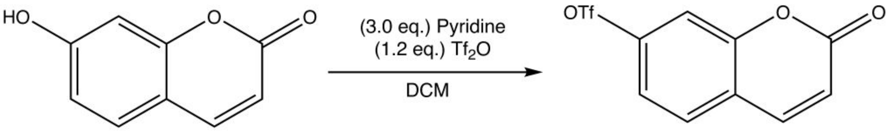

1, 62%

A suspension of umbelliferon (15.0 g, 92.9 mmol) in dichloromethane (250 mL) was cooled to 0°C, and pyridine (30 mL, 370 mmol) was added. Trifluoromethanesulfonic anhydride (19 mL, 112 mmol) in DCM (100 mL) was added dropwise over 15 min. The reaction was stirred for an additional 30 min on ice, followed by 2 hours at room temperature. The reaction was worked up by washing with brine, drying over Na_2SO_4, and the solvent removed under reduced pressure. The resulting solid was recrystallized from 1:1 ethyl acetate: hexanes yielding large off-white crystals (18.2 g, 62%). ^{1}H NMR (400 MHz, dmso) δ 8.09 (dd, J = 9.7, 1.8 Hz, 1H), 7.90 (dd, J = 8.7, 1.8 Hz, 1H), 7.69 (t, J = 2.1 Hz, 1H), 7.47 (dt, J = 8.7, 2.2 Hz, 1H), 6.57 (dd, J = 9.6, 1.8 Hz, 1H).

Scheme 2, Compound 2 – 7-azetidine-coumarin

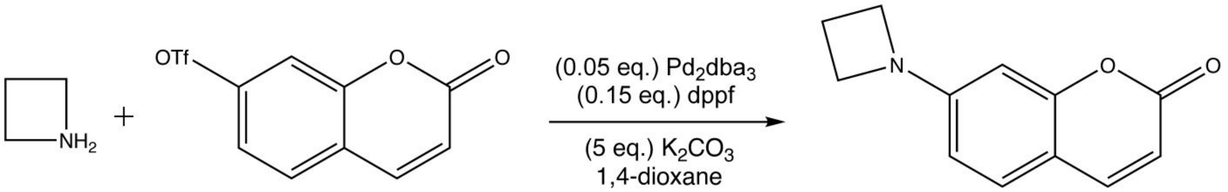

1, 62% **2**, 8%

A flask containing **1** (20.5 g, 69.8 mmol), azetidine hydrochloride (9.14 g, 97.7 mmol), Pd_2dba_3 (3.15 g, 3.5 mmol), 1,1'-Bis(diphenylphosphino)ferrocene (dppf, 5.81 g, 10.5 mmol), and potassium carbonate (48.5 g, 350 mmol) was fitted with a stir bar and purged with vacuum / nitrogen three times. Dry 1,4-dioxane was added through a cannula and suspension stirred at room temperature for 30 min. The reaction was then stirred at 80°C for 24 hours. The solution was filtered through celite and solvent removed under reduced pressure. The crude product was redissolved in 1:9 methanol: dichloromethane and filtered through a silica gel plug and extracted with 1:9 methanol: dichloromethane. This semi-pure product was then further purified by flash chromatography (methanol in dichloromethane

gradient, from 0% methanol to 2% methanol). Fractions containing product were pooled and solvent removed under reduced pressure to yield a yellow-orange powder. This was further purified by recrystallizing from ethyl acetate to afford pale yellow needle-like crystals (1.1 g, 8%). ^{1}H NMR (400 MHz, $CDCl_3$) δ 7.54 (d, J = 9.4 Hz, 1H), 7.24 (d, *J* = 8.4 Hz, 1H), 6.28 (dd, *J* = 8.4, 2.2 Hz, 1H), 6.20 (d, *J* = 2.2 Hz, 1H), 6.06 (d, *J* = 9.3 Hz, 1H), 4.00 (t, *J* = 7.4 Hz, 4H), 2.44 (p, *J* = 7.4 Hz, 2H).

Scheme 3, Compound 3 – 3-Carbaldehyde-7-azetidinylcoumarin

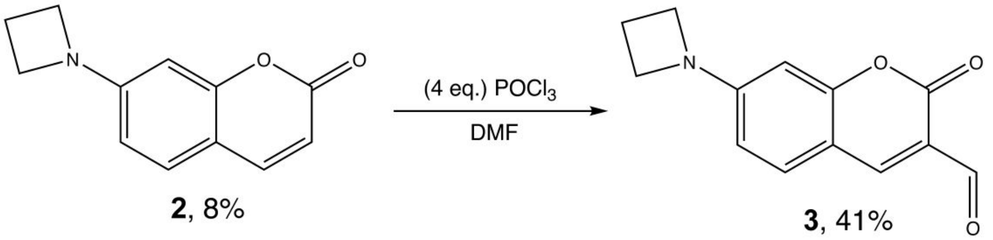

A flame-dried round bottom flask was charged with freshly distilled dimethyl formamide (10 mL), capped, and purged three times with a shallow vacuum / N_2. Phosphoryl chloride (1.4 mL, 14.8 mmol) was added dropwise, and the solution was stirred while heating at 55°C for 30 min. Compound **2** (747 mg, 3.7 mmol) dissolved in dry dimethyl formamide (10 mL) was added to activated phosphoryl chloride solution, and the reaction was stirred at 60°C for 1.5 hours. The reaction was quenched with ice and neutralized with saturated Na_2CO_3. The aqueous suspension was then extracted with dichloromethane (4x50 mL), the organic extracts dried over $MgSO_4$, and the solvent removed under reduced pressure. The crude product was purified by flash column chromatography (gradient of ethyl acetate in hexanes from 0-40%). The product from here was taken to the next step and used as is (348 mg, 41%). ^{1}H NMR (400 MHz, dmso) δ 8.74 (s, 1H), 8.21 (s, 1H), 7.58 (d, J = 8.8 Hz, 1H), 6.44 (dd, J = 8.7, 2.1 Hz, 1H), 6.27 (d, J = 2.1 Hz, 1H), 4.10 (t, J = 7.5 Hz, 4H), 2.38 (q, J = 7.6 Hz, 2H).

Scheme 4, Compound 4 (AzRP1-OH) – (E)-3-(7-(azetidin-1-yl)-2-oxo-2H-chromen-3-yl)-2-cyanoacrylic acid

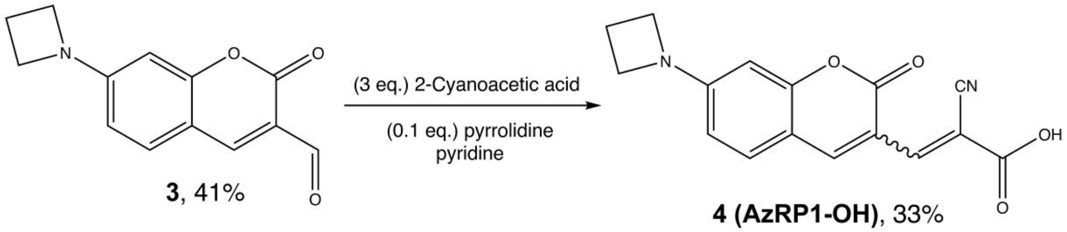

Compound **3** from the last step was taken in pyridine (50 mL), and 2-cyanoacetic acid (944 mg, 11.1 mmol) was added, followed by pyrrolidine (30 μL, 0.37 mmol). The solution was stirred overnight, giving a scarlet red suspension. Pyridine and pyrrolidine were removed

under reduced pressure, and the solid was washed with methanol, leaving behind a flaky dark red solid (150 mg, 33%). ^{1}H NMR (400 MHz, dmso) δ 8.74 (s, 1H), 8.21 (s, 1H), 7.58 (d, *J* = 8.8 Hz, 1H), 6.44 (dd, *J* = 8.7, 2.1 Hz, 1H), 6.27 (d, *J* = 2.1 Hz, 1H), 4.10 (t, *J* = 7.5 Hz, 4H), 2.39 (m, 2H).

Scheme 5, Compound 5 a, b, c (AzRP1-D a,b,c)

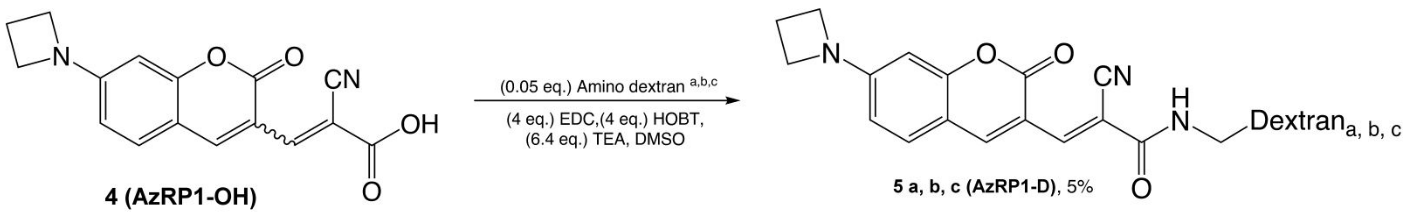

A flask was charged with Compound **4** (1.9 mg, 6.5 µmol), 1-(3-dimethylaminopropyl)-3-ethylcarbodiimide hydrochloride (4.0 mg, 26 µmol), and hydroxybenzotriazole (3.51 mg, 26 µmol), and amino dextran (10 kDa, 35 kDa, or 70 kDa, 20 amines per dextran molecule, 47.55 mg, 0.65 µmol). 5 mL of DMSO was added, and the reaction mixture was heated gently to 32 °C in a water bath to help dissolve the dextran. Triethylamine (2.6 mg, 26 µmol) was added. The reaction mixture was left to stir at room temperature for 72 hours. Once complete, the reaction mixture was quenched by pouring 5 mL of water, then purifying using a size exclusion column (Sephadex G-10) and eluting with water. Pooled fractions were dried down under a high vacuum, giving a red fluffy powder (2.37 mg, 5%). Synthesis was also confirmed using thin-layer chromatography (data not shown) and binding vs GSH (see text).

Scheme 6, Compound 6 (AzRP2-OH) – (E)-6-(3-(7-(azetidin-1-yl)-2-oxo-2H-chromen-3-yl)-2-cyanoacrylamido)hexanoic acid

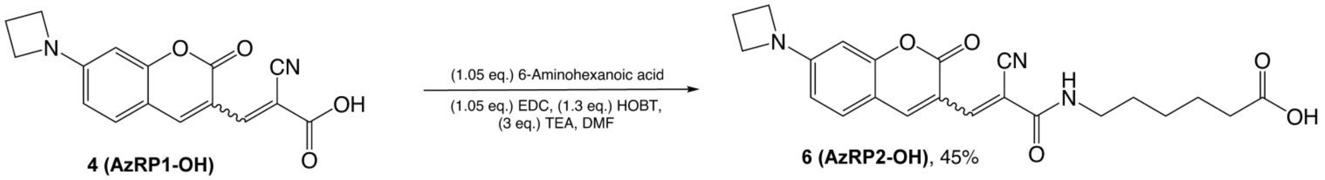

A flask was charged with Compound **4** (27 mg, 91.1 µmol), 1-(3-dimethylaminopropyl)-3-ethylcarbodiimide hydrochloride (17.5 mg, 96 µmol), and hydroxybenzotriazole (16.0 mg, 118.5 µmol) and dissolved in 5 mL of DMF. Triethylamine (38 µL, 273 µmol) was added and the reaction mixture was stirred for 1 hour for activated intermediate. 6-Aminohexanioc acid (12.5 mg, 96 µmol) was separately added to 2 mL of DMF, which was then added to the reaction mixture at once. The solution was stirred for 16 hours until complete. The reaction mixture was concentrated under reduced pressure and purified by flash column chromatography (gradient of methanol in dichloromethane, 0% to 5%), leaving a dark red powder (16.5 mg, 45%).^{1}H NMR (400 MHz, DMSO, major isomer) δ 8.66 (t, *J* = 5.7 Hz, 1H), 7.95 (s, 1H), 7.44 (d, *J* = 8.7 Hz, 1H), 7.36 (s, 1H), 6.35 (dd, *J* = 8.6, 2.1 Hz, 1H), 6.21 (d, *J* =

2.1 Hz, 1H), 4.01 (t, *J* = 7.4 Hz, 4H), 3.09 (q, *J* = 6.6 Hz, 2H), 2.37 (m, 2H), 2.05 (t, *J* = 7.5 Hz, 2H), 1.37 (m, 4H), 1.25 (m, 2H).

Scheme 7, Compound 7 a, b, c (AzRP2-D a,b,c)

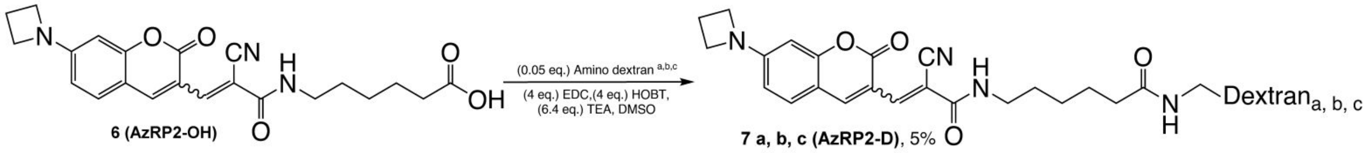

A flask was charged with Compound **6** (3.2 mg, 7.8 µmol), 1-(3-dimethylaminopropyl)-3-ethylcarbodiimide hydrochloride (6 mg, 31 µmol), hydroxybenzotriazole (4.2 mg, 31 µmol), and amino dextran (10 kDa, 35 kDa, or 70 kDa, 20 amines per dextran molecule, 26.5 mg, 0.39 µmol). 5 mL of DMSO was added, and the reaction mixture was heated gently to 32 °C in a water bath to help dissolve the dextran. Triethylamine (5.1 mg, 50 µmol) was added. The reaction mixture was left to stir at room temperature for 72 hours. Once complete, the reaction mixture was quenched by pouring 5 mL of water, then purifying using a size exclusion column (Sephadex G-10) and eluting with water. Pooled fractions were dried down under a high vacuum, giving a red fluffy powder (1.325 mg, 5%). Synthesis was also confirmed by thin-layer chromatography (Not shown), and binding vs GSH (see text).

Scheme 8, Compound 8 (AzRP1-Morph) – 3-(7-(azetidin-1-yl)-2-oxo-2H-chromen-3-yl)-2-cyano-N-(2-morpholinoethyl)acrylamide

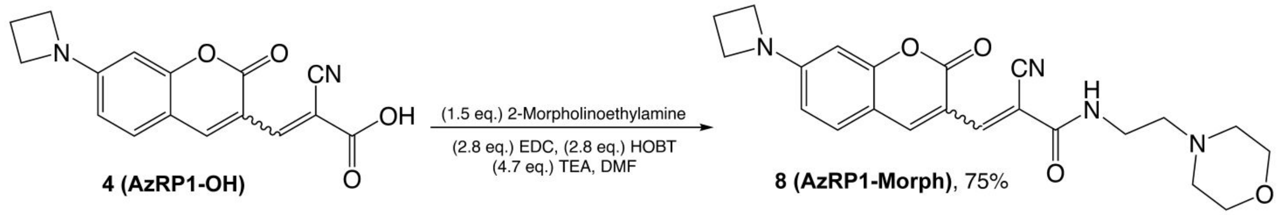

A flask was charged with Compound **4** (3.13 mg, 10.6 µmol), 1-(3-dimethyl aminopropyl)-3-ethyl carbodiimide hydrochloride (6 mg, 30 µmol), and hydroxy benzotriazole (4.2 mg, 30 µmol), and 2-morpholinoethylamine (2.06 mg, 16 µmol). 500 µL of DMF was added, followed by the addition of triethylamine (5 mg, 50 µmol), and the reaction mixture was stirred covered in foil. After 16 hours, the reaction mixture was taken in DCM (30 mL) and partitioned with 150 mL saturated $NaHCO_3$. The aqueous layer was extracted with an additional 30 mL of DCM twice. The combined organic extracts were extracted with 40 mL of 0.1N HCl 4 times. Finally, the aqueous extracts were neutralized with saturated $NaHCO_3$ and extracted with DCM until the aqueous layer was colorless (50 mL 4 times). The extract was dried over $MgSO_4$, then the solvent was removed under reduced pressure to afford AzRP1-Morph as a red residue (2.98 mg, 75% yield). ^1H NMR (400 MHz, $CDCl_3$, major isomer) δ 8.69 (s, 1H),

8.54 (s, 1H), 7.72 (s, 1H), 7.38 (d, J = 9.0 Hz), 6.31 (d, J = 9.3 Hz, 1H), 6.14 (s, 1H), 4.11 ((t, J = 7.5 Hz, 4H), 3.74 (m, 8H), 3.49 (m, 2H), 2.53 (m, 2H), 2.51 (m, 2H).

Figure S2.1) Compound 1 – ^{1}H-NMR

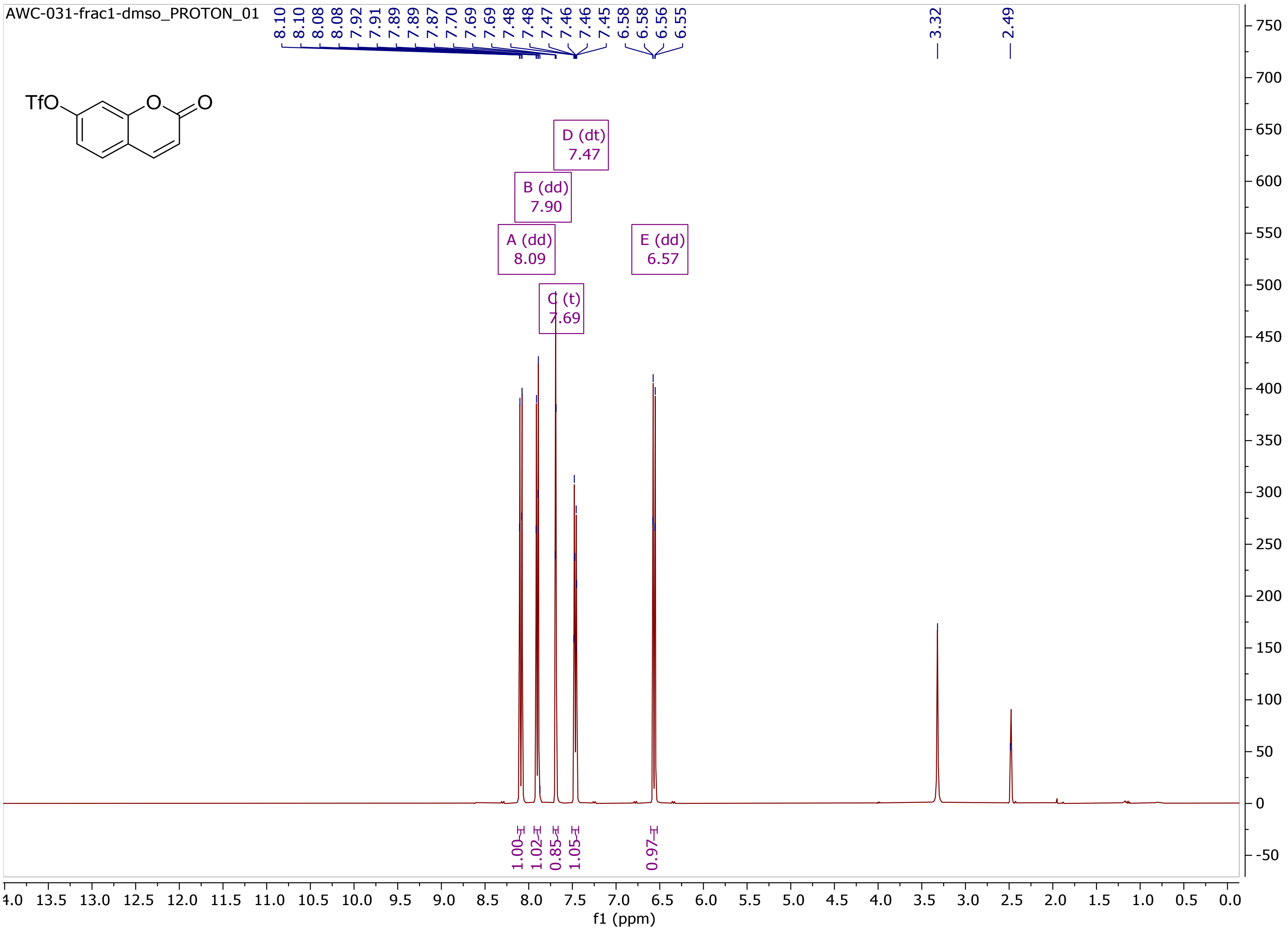

Figure S2.2) Compound 2 – ^{1}H-NMR

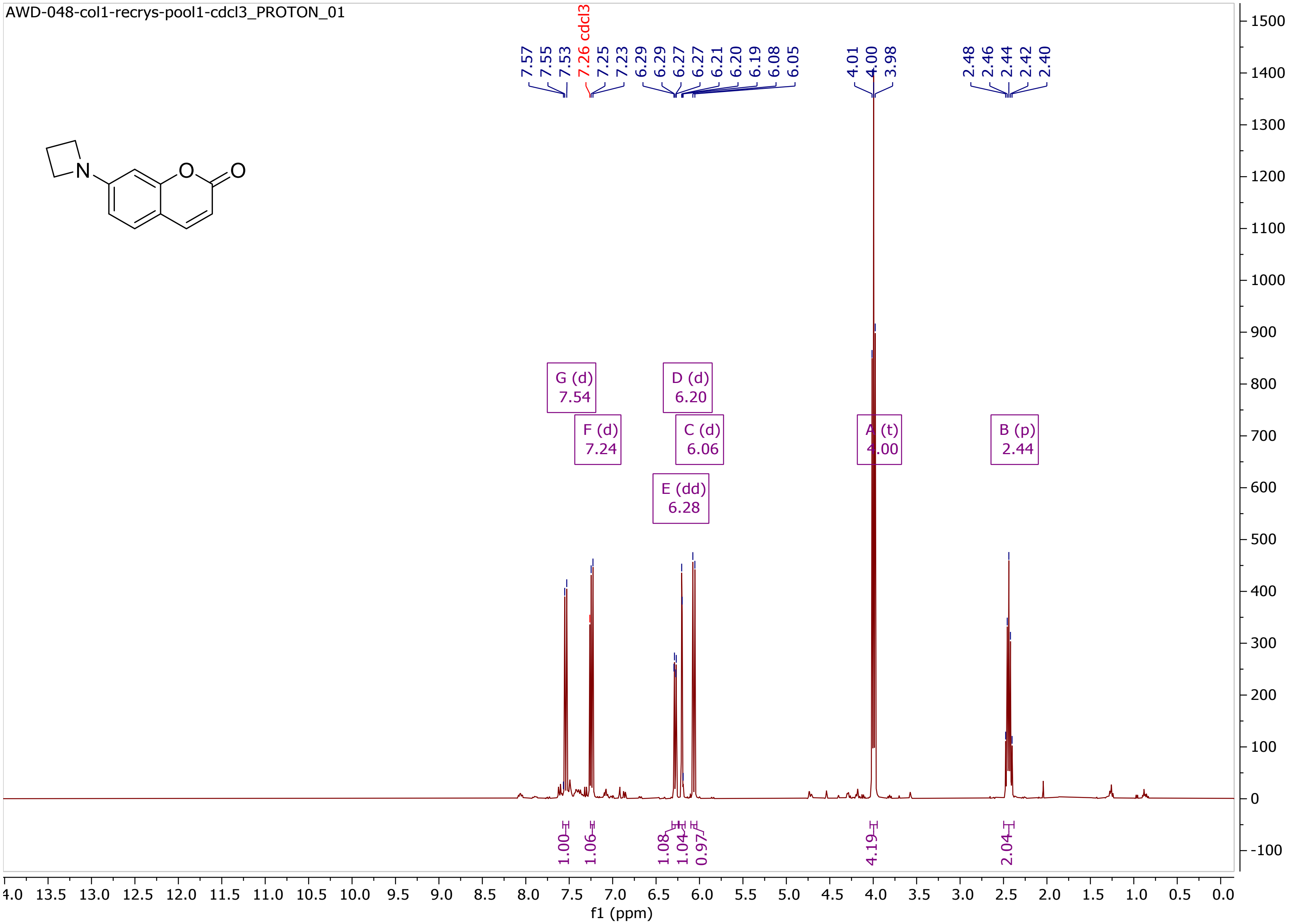

Figure S2.3) Compound 3 – ^{1}H-NMR

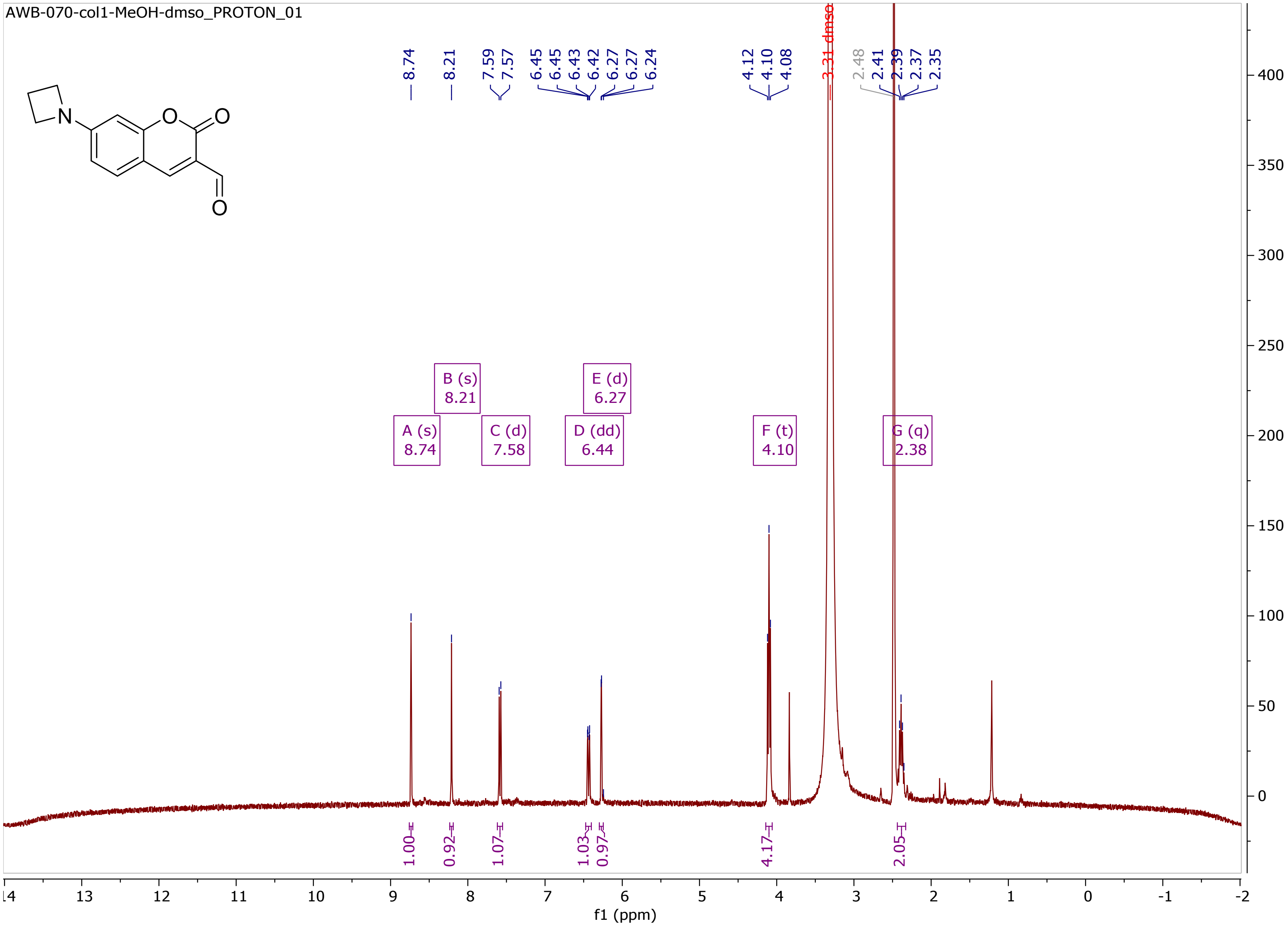

Figure S2.4) Compound 4 (AzRP1-OH) – ^{1}H-NMR

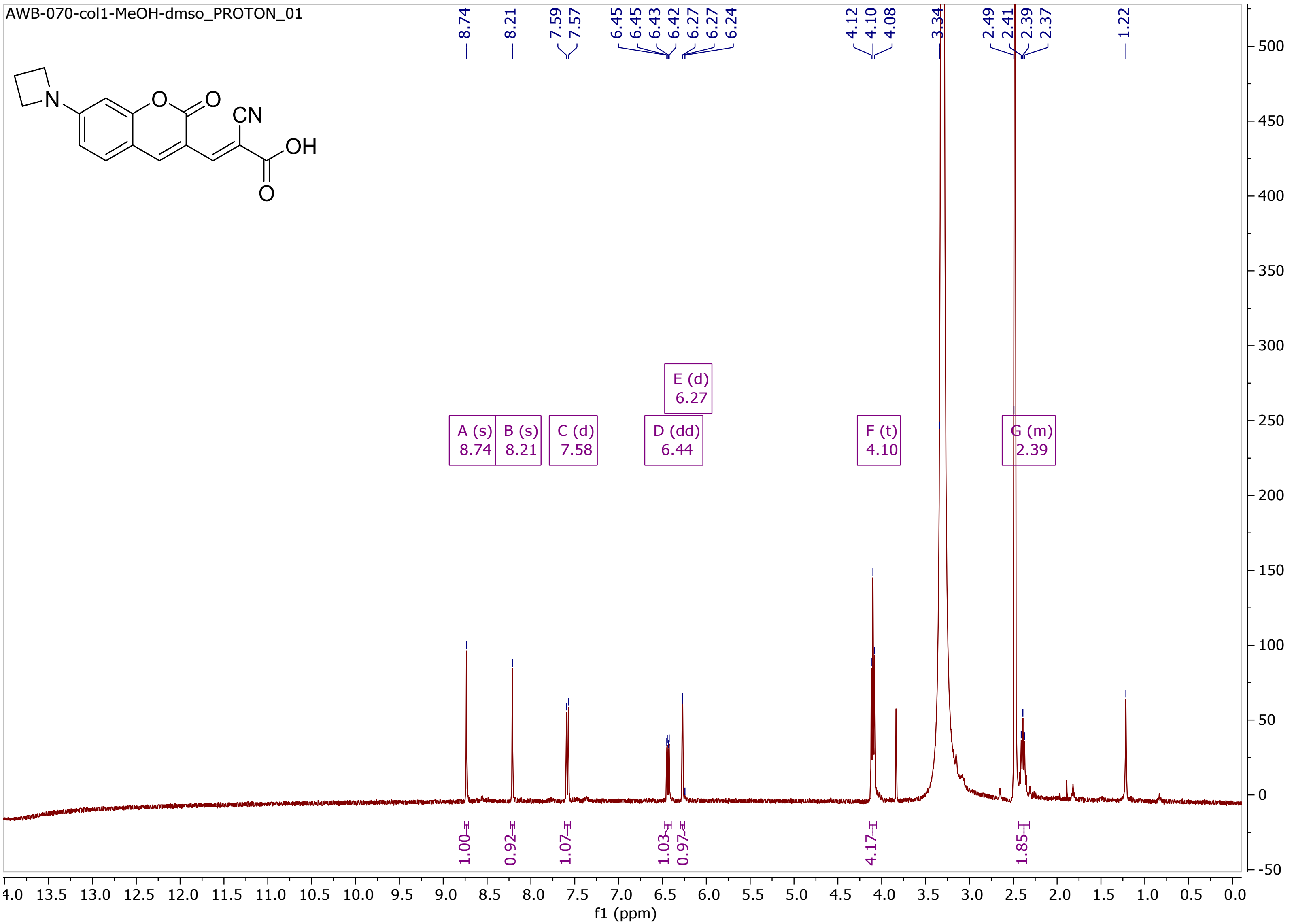

Figure S2.5) Compound 5c (AzRP1-D 70kDa)

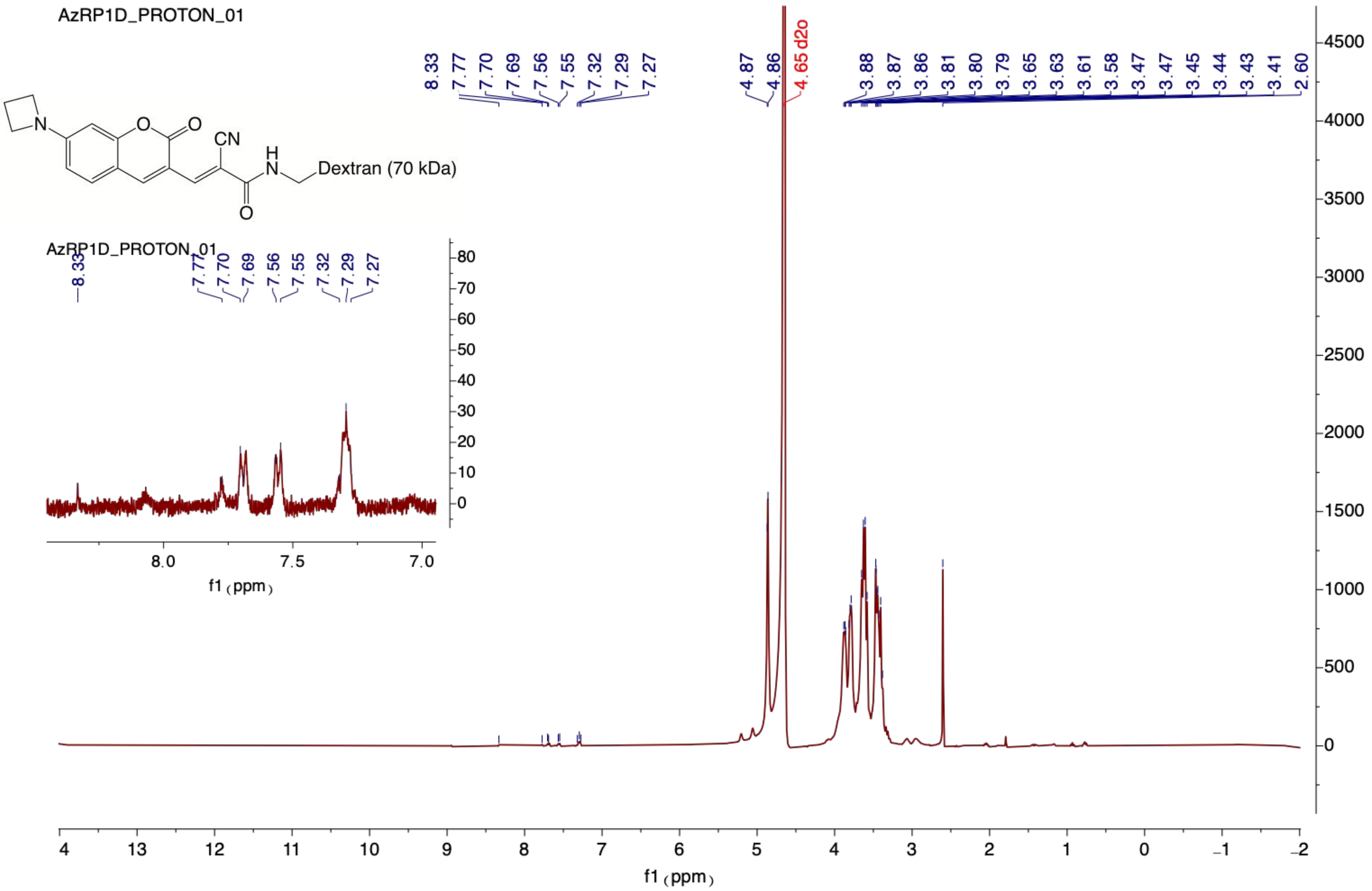

Figure S2.6) Compound 6 (AzRP2-OH) – ^{1}H-NMR

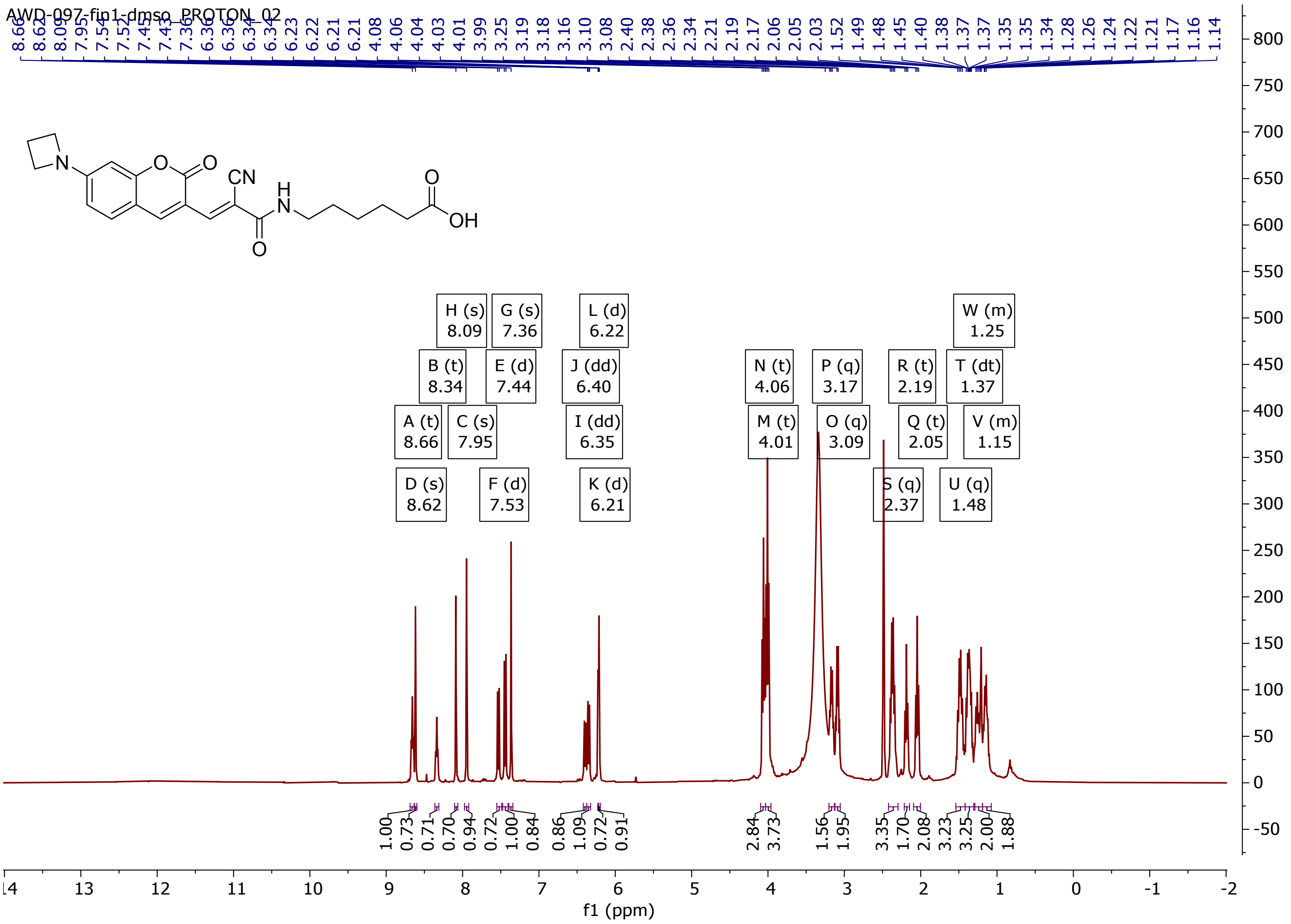

Figure S2.7) Compound 6 (AzRP2-OH) – gCOSY NMR

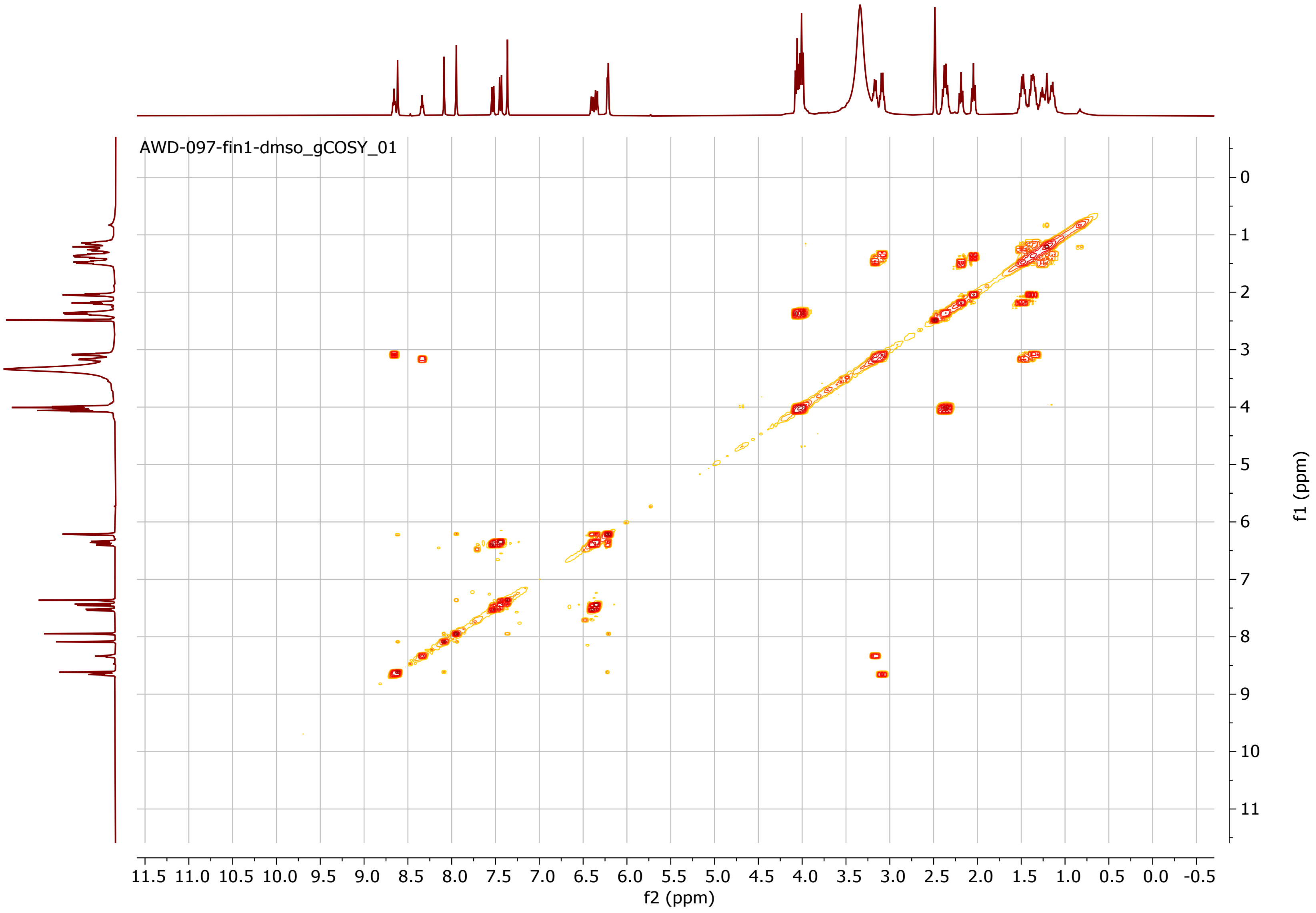

Figure S2.8) Compound 7c (AzRP2-D 70kDa) – ^{1}H-NMR

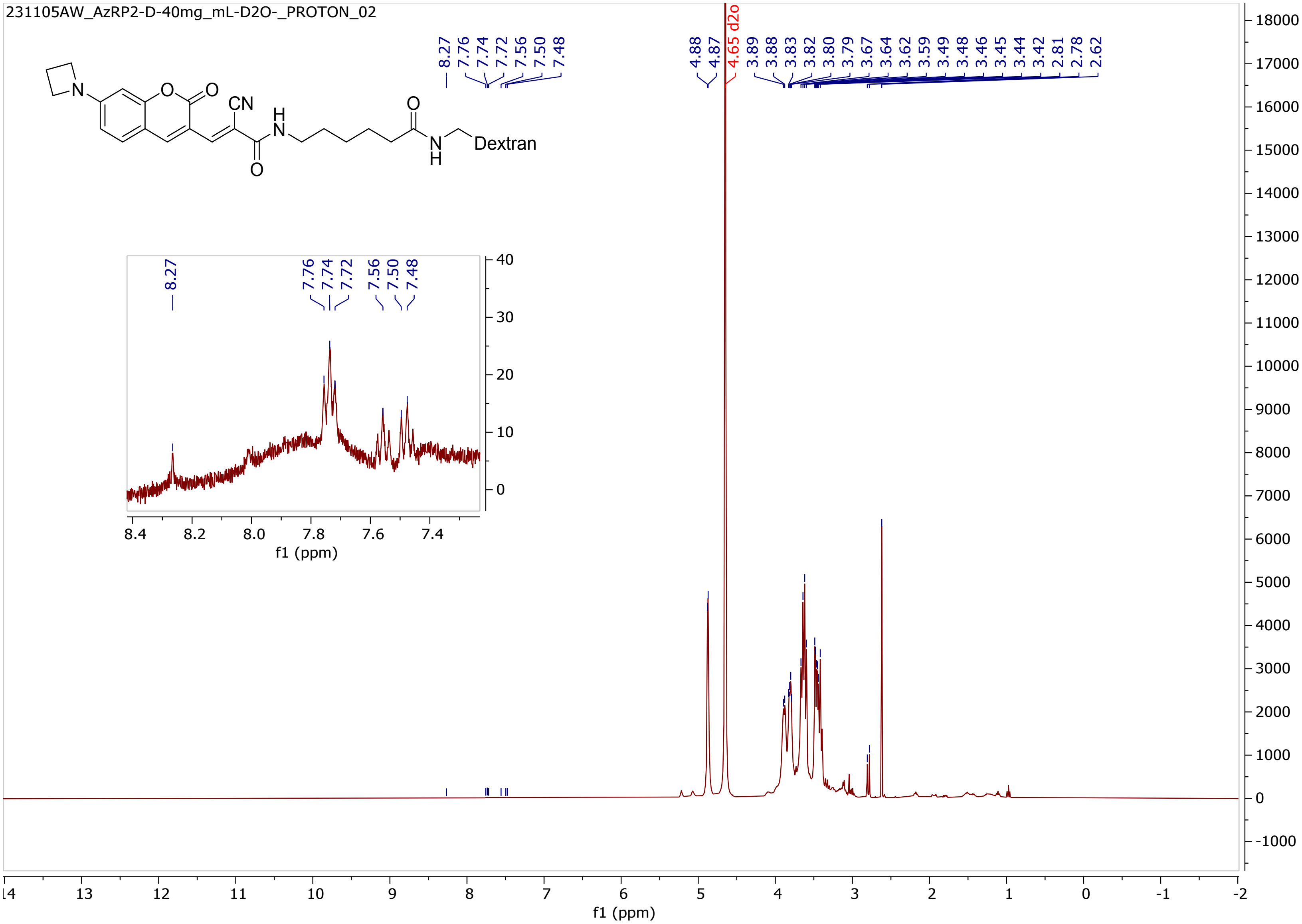

Figure S2.9) Compound 8 (AzRP1-Morph) – ^{1}H-NMR

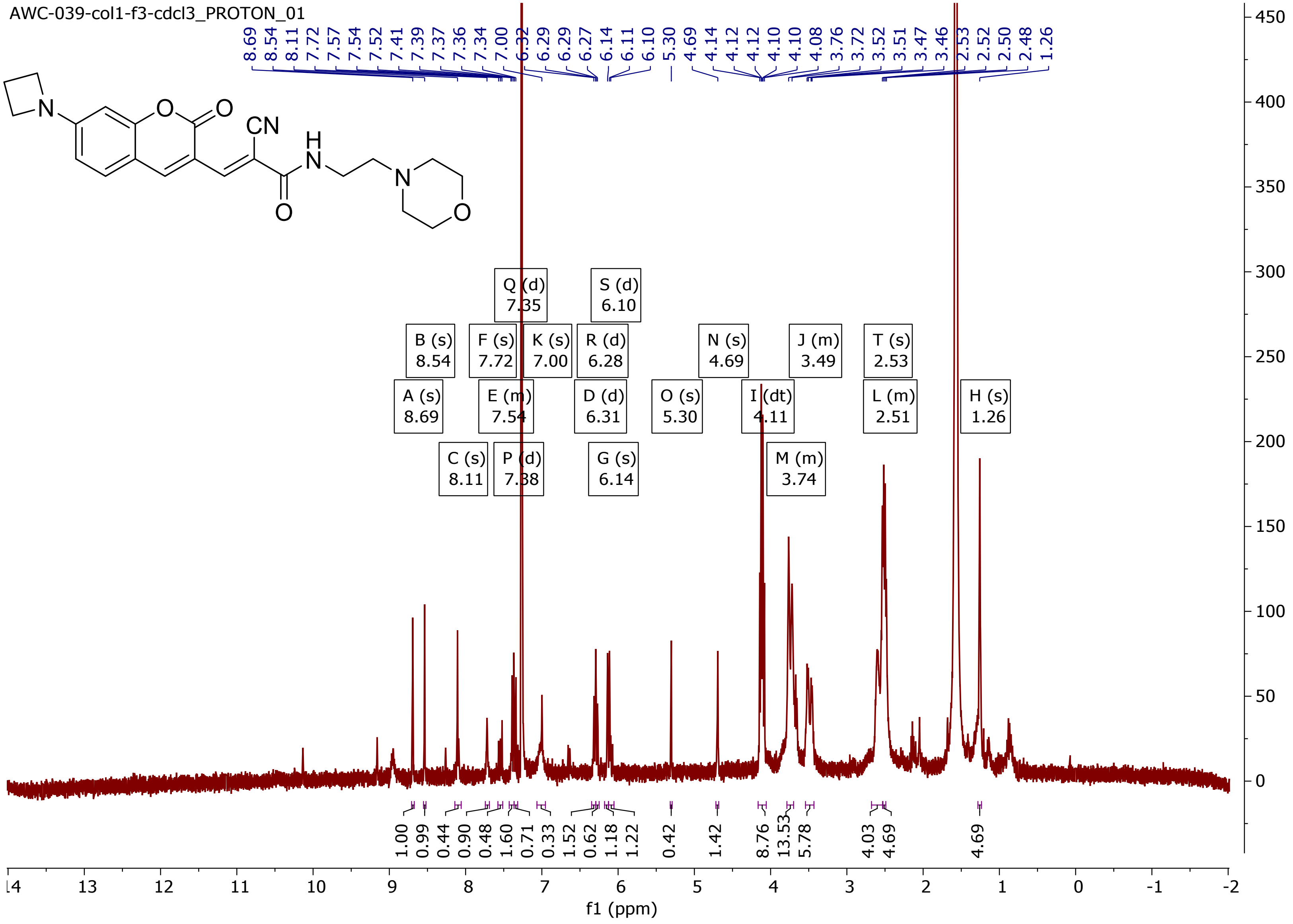

2) Figure S3 Absorbance of Compound 6 (AzRP2-OH) vs Compound 7c (AzRP2-Dc)

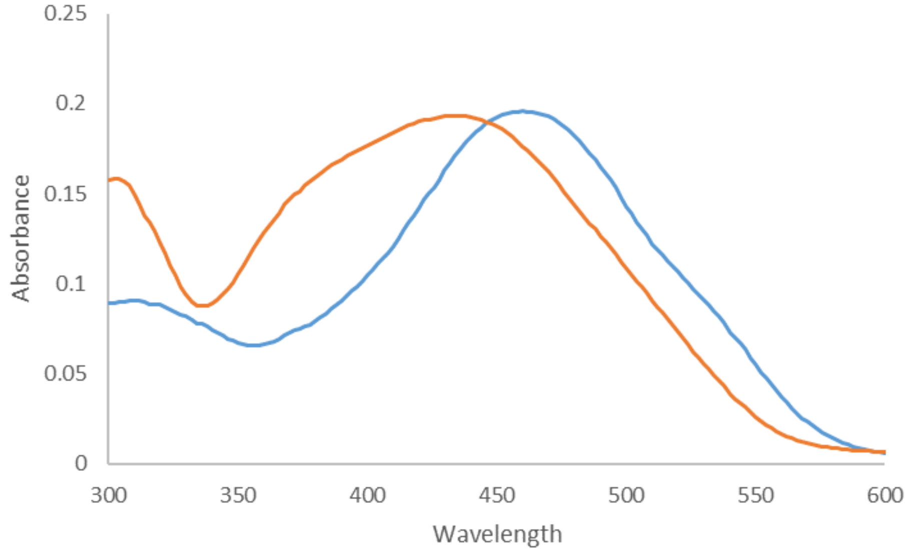

Figure S3 Absorbance of 5.5 µM compound **6** (AzRP2-OH, orange line) and 5.5 µM **7c** (AzRP2-Dc). Based on Abs_{max} for the respective probes, the molar stoichiometry of the probe to dextran in **7c** is essentially 1:1.

3) Figure S4 X-ray crystal structures of CAS 54711-39-6 and Compound 3

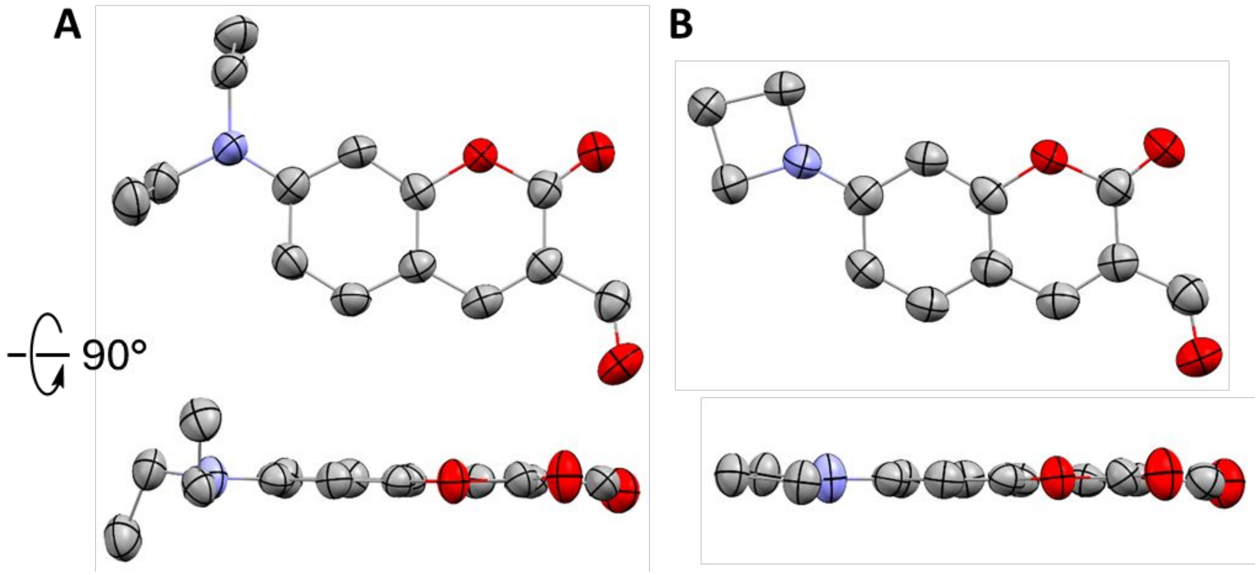

Figure S4 Crystal structures of A) 7-diethyl **(CAS 54711-39-6)** and B) 7- azetidine 3- formyl coumarin (**3;** cf. Fig. 1) fluorophores. Ellipsoid shapes indicate 95% electron density about individual atoms. No clearly discernable difference in electron density easily explains vastly higher QE for the azetidine derivative[35-37]; Fig. 1), suggesting a P-orbital orientation explanation for enhanced QE is likely (see text). See Table S1 for crystallography data.

4) Figure S5 Optimized optical path; characteristics of a customized filter cube used in widefield microscopy (SCP) experiments vs cube characteristics used previously by others

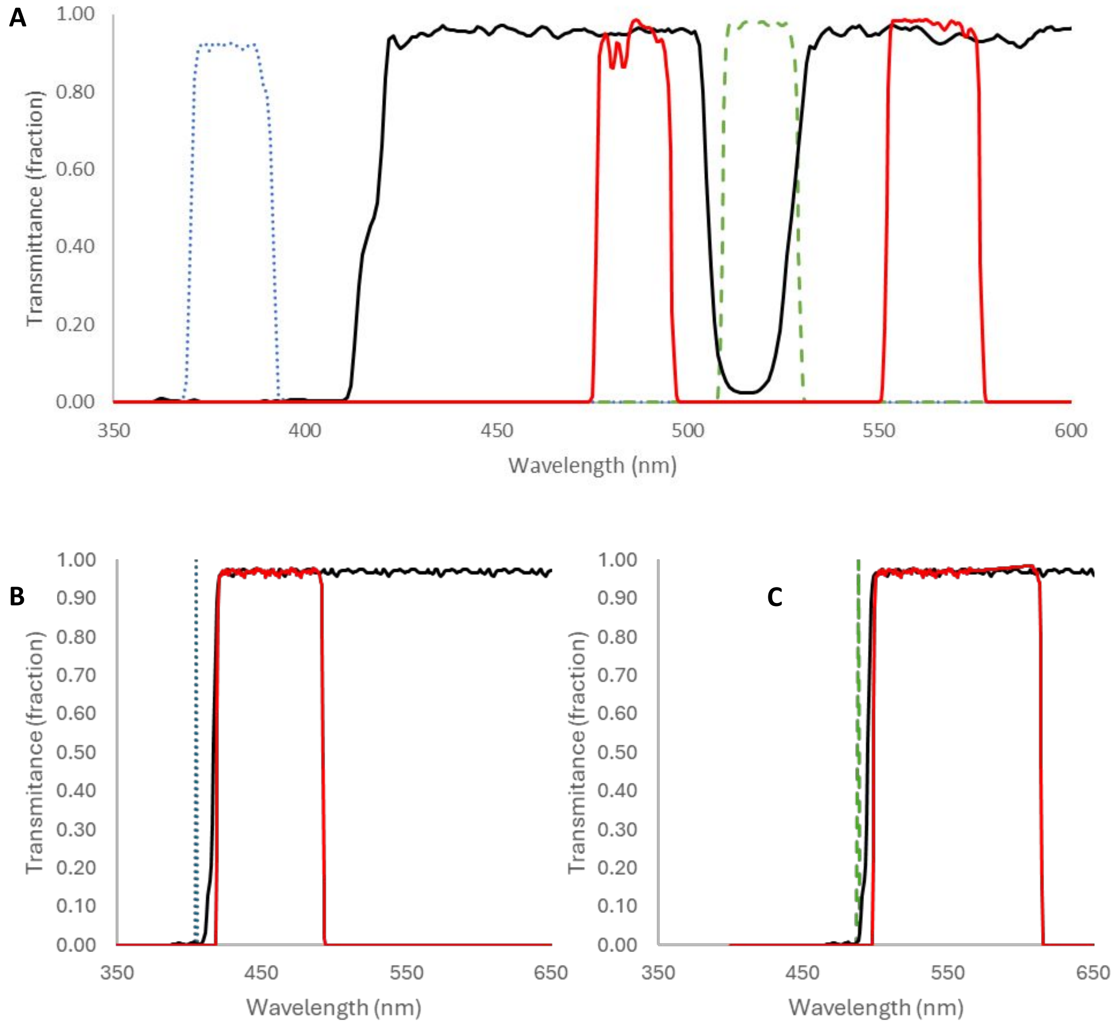

Figure S5 Optical characteristics of a customized widefield fluorescence filter cube and filter configuration used to collect ratiometric fluorescence data from live malarial parasites in this work (top, A) vs the laser scanning confocal microscope (LSCM) optical configurations used to measure azetidinyl coumarin acetoxymethyl ester probe in HeLa cells or neurons in earlier work by Jiang et al. (bottom, B and C; [34,35]). In A, the dotted blue line represents 385 nm-centered transmittance (low wavelength excitation), the dashed green is 520 nm-centered transmittance (high wavelength excitation), and the solid red lines show emission band filters centered at 465 nm +/- 10 nm and 565 nm +/- 10 nm. The solid black line denotes wavelengths reflected by the dichroic mirror positioned within the cube (emission is reflected towards the CCD camera [Fig. S3]). In this configuration the dichroic mirror within the custom cube positioned underneath the epifluorescence microscope stage reflects any light below 410 nm and transmits longer wavelength light from 411-505 nm (solid black) that is passed through an emission band filter (red line) on to a CCD camera, reflects light again from 506-525 nm, and also transmits a second band of light ≥ 526nm. The dual-band pass emission filter configuration positioned within the dichroic mirror is used to collect emission intensities from 475-495 nm and 555-575 nm ([solid,red line]. 20 nm bandwidth for each band, corresponding to peak emission bands +/- 10 nm. As described in the text, two separate excitation filters were also placed in a motorized filter wheel in front of a Xenon arc lamp housing, one 385 nm (dashed blue, 20 nm bandwidth) and one 520 nm (dashed green, 20 nm bandwidth). A third filter disk (blanking all light made of solid aluminum) was used in between excitation at either wavelength to prevent bleeding between the two channels and as a step in the computer-controlled data collection cycle between excitation bands to allow for recovery from photobleaching. B and C) Measurements made by Jiang *et al* [34,35] used two separate filter cubes to collect fluorescent images from cells under laser illumination. B) The GSH bound probe (RT-GSH) was excited with a 405 nm laser (blue dotted line) of unknown power reflected by a 410 nm dichroic mirror (black solid line), which reflects the laser light and transmits emission light at a longer wavelength. A 418-495 nm filter (red solid line) was used to collect fluorescence emission. C) The free probe ("RT", green, Fig. 4 [35]) was detected by illuminating with a 488 nm laser (green dashed line) of unknown power reflected by a 495 nm dichroic mirror (solid black line), which reflects the laser light and transmits light at longer wavelength, and a much wider band 499-615 nm emission filter (solid red line) was used to collect RT fluorescence emission.

5) Figure S6 SCP schematic

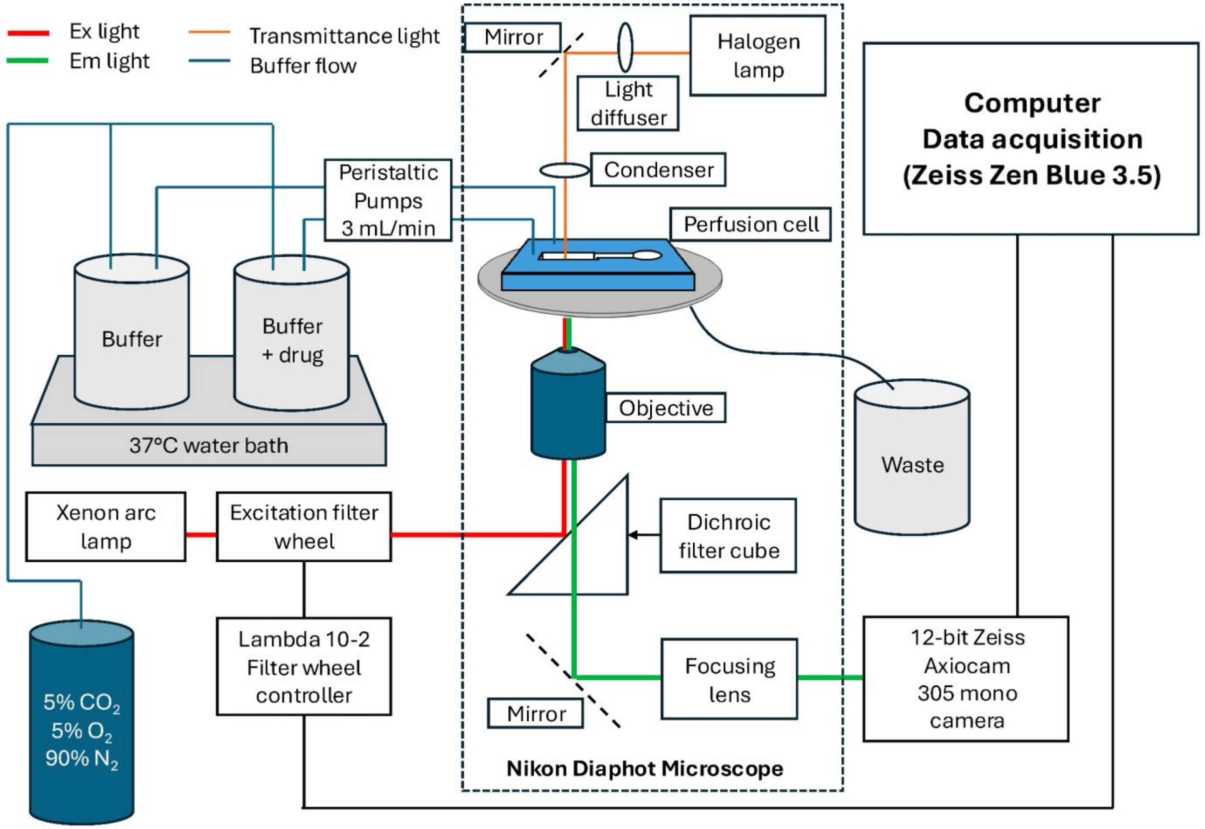

Figure S6 Schematic diagram of the SCP system used here. Perfusion buffers are continuously kept at 37°C and also continuously purged with 5% CO_2 and 5% O_2, balance N_2. The excitation light from a Xenon arc lamp is passed through a computer-controlled excitation filter wheel which then leads to a liquid light guide that guides excitation light to the microscope base (red line). A dual – excitation / dual – emission filter cube guides excitation light of appropriate wavelength to the sample. Emission light is focused onto a Zeiss Axiocam 305 mono (green line) and photons are quantified within ROI of fixed size by Zeiss Zen Blue 3.5 on the controller / acquisition computer.

6) Figure S7 Effect of pH on Compound 7c (AzRP2-Dc) GSH – dependent quasi fluorescence

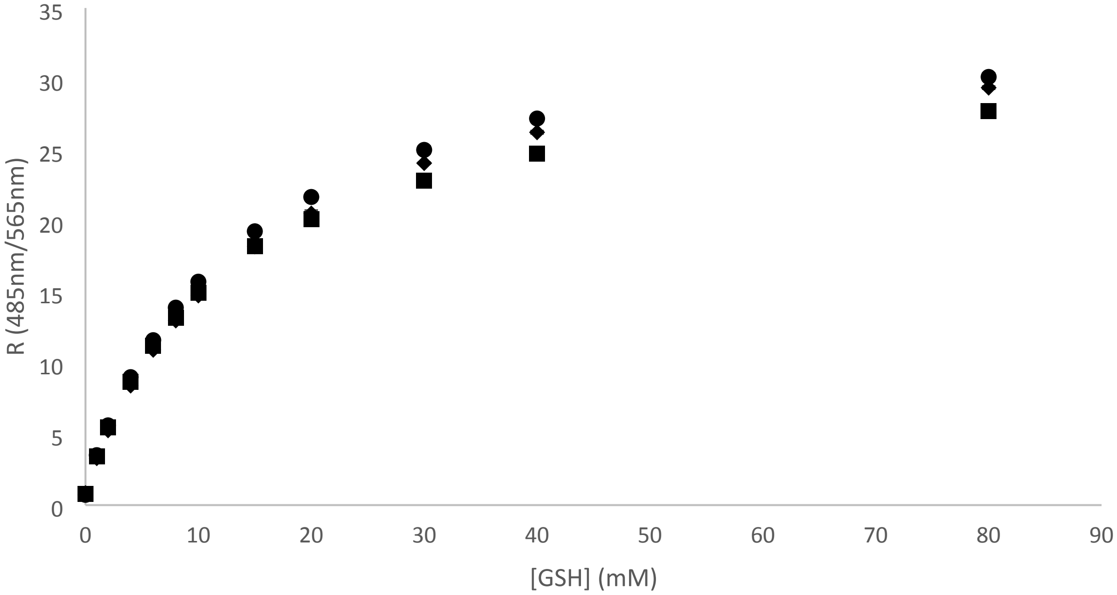

Figure S7 Compound **7c** (AzRP2-Dc) quasi ratiometric probe response to [GSH] vs pH. At 3 different pH (5.0, 6.0, 7.0) (circles, squares, diamonds, respectively) there is no significant change in probe response vs [GSH]. Solutions were made in either 0.1M sodium propionate (pH 5.0, 6.0) or 0.1M sodium phosphate (pH 7.0) buffers. For each solution, 200 µL at each [GSH] and pH, with a constant probe concentration of 2 µM, was added in triplicate to wells of a 96-well plate. Measurements were made using a Tecan fluorescence plate reader using alternate excitation at 385 nm and 520 nm, followed by ratioing emission at 485 nm and 565 nm, respectively. Excitation bandwidth was 9 nm, emission bandwidth was 20 nm, and the values reported are the average ± SEM (Note: SEM is too small to observe visually as it falls within the symbols).

7) Figure S8 Calibration of 7c probe fluorescence vs concentration and vs [GSH]

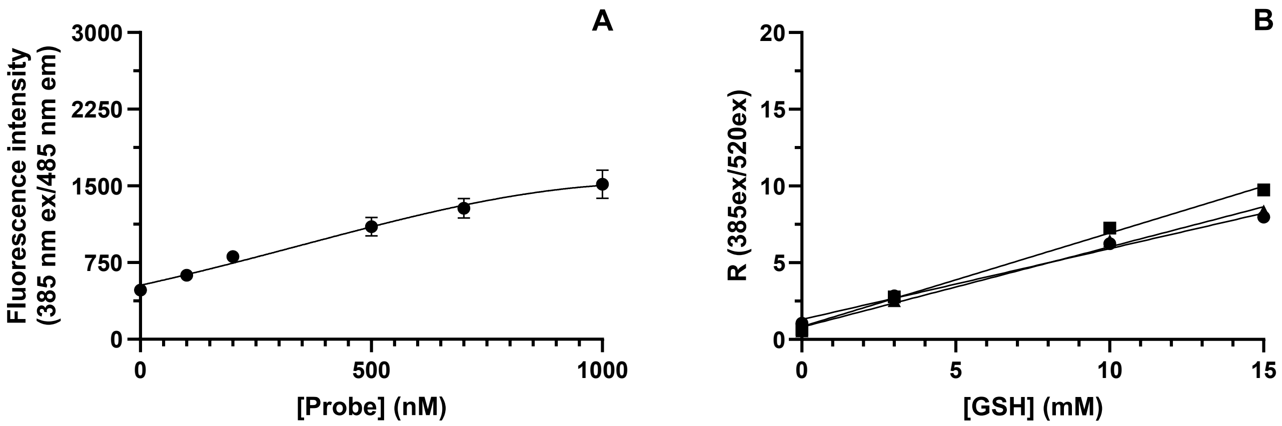

Figure S8A-B) Compound **7c** (AzRP2-D) fluorescence vs. [probe] **(A, left)** vs [GSH] at 3 [probe] **(B, right) A)** Thin layer calibration of **7c** (AzRP2-Dc) fluorescence emission using widefield microscopy (Fig. S3,S4) exactly as applied to live parasites, in the absence of GSH at various probe concentrations, measured using 385 nm excitation as described in methods. The quadratic fit has the equation $FI=516.2*1.198*[Probe]-0.0004233*[Probe]^2$ with R^2 of 0.92. **B)** GSH – dependent ratios of 485 nm / 565 nm fluorescence emission intensity for Compound **7c** (AzRP2-D) is essentially concentration independent across 2 orders of magnitude of probe concentration (circles; 200 nM, squares; 2 μM, triangles; 20 μM). Ratios were measured using a fluorescence plate reader and are 385 nm excitation / 485 nm emission divided by 520 nm excitation / 565 nm emission as described in Methods. Data shown are the average of 3 measurements, each in triplicate; error bars are +/- S.D. but are smaller than data symbols, so they cannot be easily seen.

8) Figure S9 Extended data sets for 7c fluorescence vs [GSH] and quadratic fits

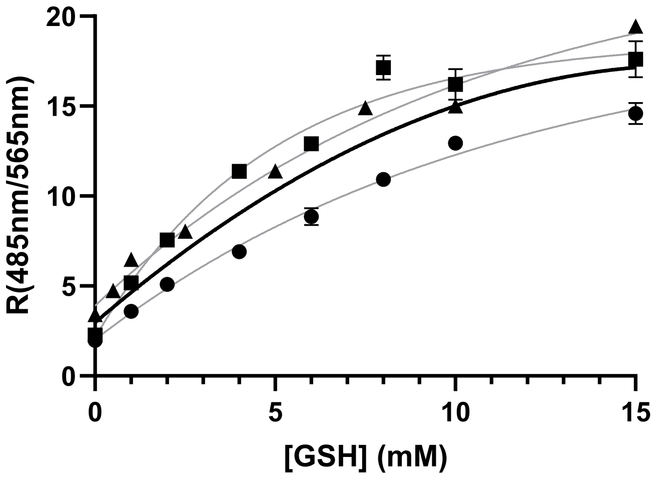

Figure S9 Thin layer calibration of **7c** (AzRP2-Dc) vs GSH using SCP identical to that used for live cells. The extended data set showed overlapping quadratic fit for multiple [probe]. Ratiometric titration of **7c** (485 nm/565 nm emission upon alternate 385 nm/520 nm excitation, see Fig. S3, S4) at 3 different probe concentrations that span expected DV concentrations of probe, circles: 200 nM probe; squares: 500 nM probe; triangles: 1 µM probe. Grey lines are quadratic fits to individual data sets at one [probe] with R^2 of 0.99 (200 nM), 0.97 (500 nM), and 0.99 (1 µM) for each concentration. The average quadratic fit of all three GSH titrations is shown as a heavy black line with equation $R=2.966+1.722*[GSH]*-0.05178*[GSH]^2$ (R^2=0.90 using all data points).

9) Figure S10 Fluorescence spectra of Compound 7c (AzRP2-D) +/- GSH, +/- H_2O_2)

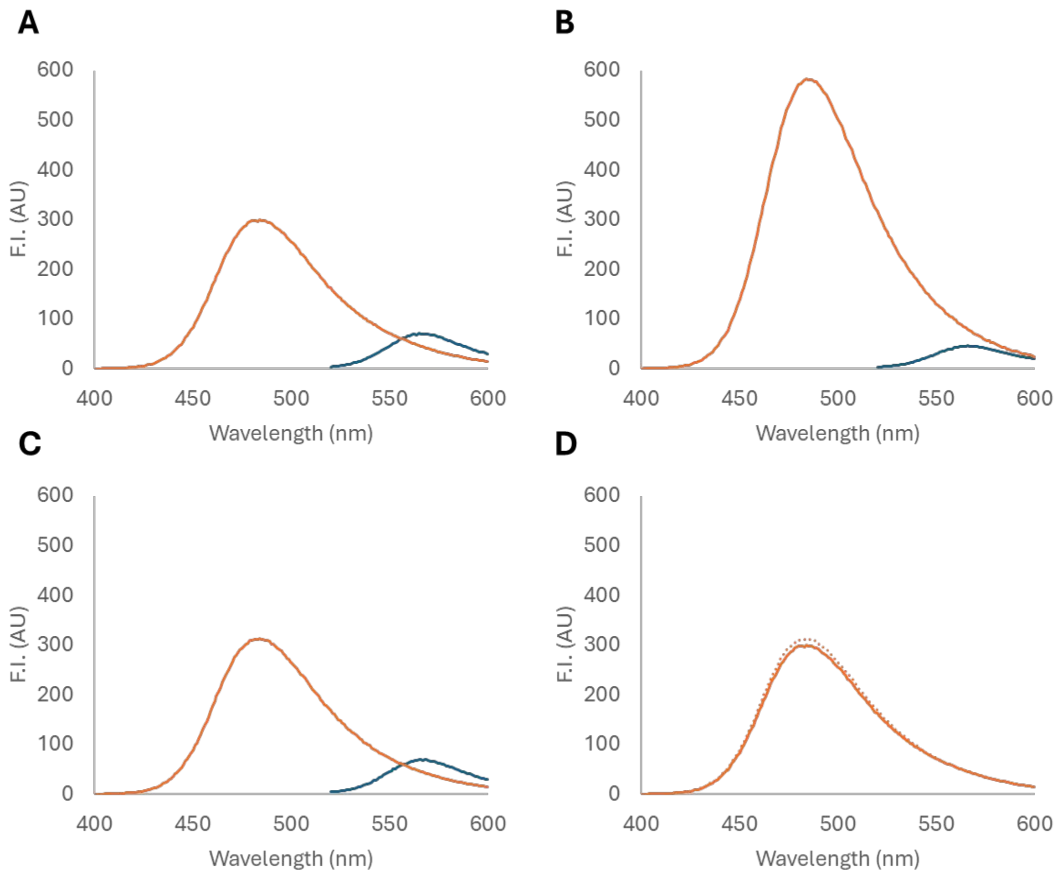

Figure S10 A-C) Fluorescence power spectra at 385 nm excitation (orange) or 520 nm excitation (blue) of A) 1 µM **7c** in pH 5.2 propionate buffer in the absence of GSH, B) 1 µM **7c** incubated with 3 mM GSH for 10 min., C) 1 µM **7c** incubated with 3 mM GSH for 10 min, followed by incubation with 5 mM H_2O_2 for 10 min. D) Emission spectra after 385 nm excitation of A) (solid line) vs C) (dotted line) corresponding to the conditions measured in the complete oxidation of parasite DV samples (Fig. 8), illustrating that exposure to 5 mM H_2O_2 completely disassociates any GSH bound to **7c** probe as it then oxidizes the GSH in solution to GSSG.

10) Figure S11 Distribution of $[GSH]^{DV}$ found for the 4 different *P. falciparum* strains

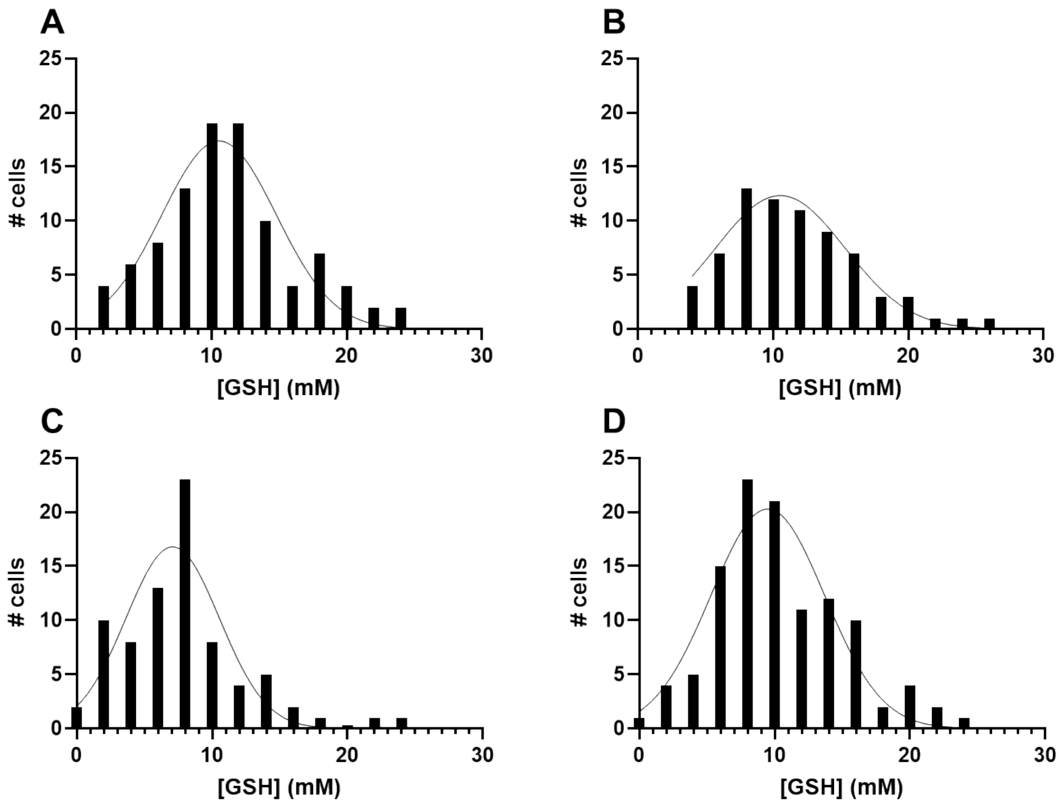

Figure S11 Histogram showing the distribution of $[GSH]^{DV}$ found for the 4 different *P. falciparum* strains examined in this work (> 72 cells, each strain). The CQS, ARTS reverse-engineered strain C2[GC03] (A) and its CQR, ARTS partner strain C4[Dd2] (B) are the progeny of allelic exchange, differing only in the *pfcrt* gene they express, either that encoding the GC03 (CQS) or Dd2 (CQR) isoform, respectively[41]. The CQR, ARTS strain CamWT (C) and reverse engineered CQR, ARTR strain CamWT[K13-C580Y] (D) differ only at a single codon in the *pfk13* gene, which leads to a C580Y substitution in the encoded PfK13 protein[42]. Frequency distributions shown are with a binning width of 2 mM for all samples and are fitted to a smooth Gaussian curve that overlays each histogram (thin solid lines). Averages are shown in Table 1

11) Figure S12 Effects of drugs alone on Compound 7c (AzRP2-Dc) fluorescence

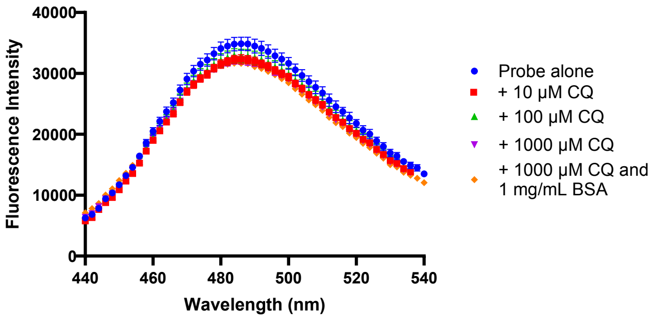

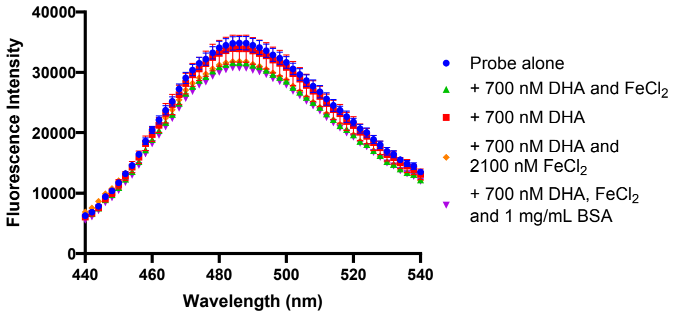

Fig. S12 Lack of drug-alone effect on probe fluorescence. A light-protected tube of **7c** was pre-incubated with 10x GSH for at least 30 minutes and aliquots mixed with variable [DHA] (top) or [CQ] bottom. Top, 100 µL of the incubated GSH-saturated probe solution was pipetted into a black-walled clear bottom 96-well plate. Next, no drug (blue circles) or 700 nM DHA alone (red squares) or DHA + 2100 nM (orange diamonds), or 700 nM (green triangles) Fe(II)Cl_2 to activate DHA or DHA + 700 nM Fe(II)Cl_2 and 1 mg/mL BSA (to mimic activation of DHA within a DV proteinaceous interior; purple triangles) were added to the respective wells. Final volume of each well = 200 µL and samples were mixed by pipetting gently (top panel). Bottom, probe was mixed with buffer + / - 1 mg/mL BSA, and CQ was added such that the final [CQ] was 10 (red squares), 100, (green triangles) or 1000 (purple triangles and orange diamonds) µM, respectively, again final volume for each well was 200 µL. All samples were prepared in 0.1M propionate buffer (pH 5.0) and measured in triplicate. The fluorescence emission spectra were collected < 10 min post-drug addition with 385 nm excitation on a Tecan infinite M200 plate reader (Männedorf, Switzerland). Top read measurements were conducted with excitation and emission bandwidths of 9 and 20 nm, respectively. Each data point is the average of 3 individual measurements from 3 samples in 3 plate wells +/- S.D. error bars overlap for the different samples in both top and bottom panels and so may not be fully visible.

12) Table S1-S3 crystallographic data for CAS 54711-39-6 and Compound 3

Table S1 Crystallographic data for **CAS 54711-39-6** and Compound **3**

	**CAS 54711-39-6**	Compound **3**
Identification code	t4_14_a	20091_a
Empirical formula	C14 H15 N O3	C13 H11 N O3
Formula weight	245.27	229.23
Temperature	100(2) K	100(2) K
Wavelength	0.71073 Å	0.71073 Å
Crystal system	Triclinic	Triclinic
Space group	P-1	P-1
Unit cell dimensions	a = 7.0632(10) Å α= 87.417(2)°.<br>b = 7.3060(11) Å β= 86.061(2)°.<br>c = 12.5430(19) Å γ = 66.773(2)°.	a = 6.128(2) Å α= 95.481(7)°.<br>b = 9.000(3) Å β= 104.416(7)°.<br>c = 9.712(4) Å γ = 92.208(7)°.
Volume	593.29(15) Å^3	515.3(3) Å^3
Z	2	2
Density (calculated)	1.373 Mg/m^3	1.477 Mg/m^3
Absorption coefficient	0.097 mm^{-1}	0.106 mm^{-1}
F(000)	260	240
Crystal size	0.319 x 0.104 x 0.063 mm^3	0.326 x 0.082 x 0.064 mm^3
Theta range for data collection	3.035 to 28.398°.	2.178 to 25.475°.
Reflections collected	9673	11037
Independent reflections	2971 [R(int) = 0.0262]	1908 [R(int) = 0.0443]
Completeness to theta = 25.242°	99.6 %	99.9 %
Absorption correction	Semi-empirical from equivalents	Semi-empirical from equivalents
Max. and min. transmission	0.746069 and 0.729215	0.99640 and 0.97575
Refinement method	Full-matrix least-squares on F^2	Full-matrix least-squares on F^2
Data / restraints / parameters	2971 / 0 / 165	1908 / 0 / 154
Goodness-of-fit on F^2	1.024	1.022
Final R indices [I>2sigma(I)]	R1 = 0.0386, wR2 = 0.0979	R1 = 0.0408, wR2 = 0.1001
R indices (all data)	R1 = 0.0564, wR2 = 0.1079	R1 = 0.0634, wR2 = 0.1113
Extinction coefficient	n/a	n/a
Largest diff. peak and hole	0.387 and -0.195 e.Å^{-3}	0.228 and -0.245 e.Å^{-3}

Table S2. Atomic coordinates (x 10^4) and equivalent isotropic displacement parameters ($Å^2$x 10^3) for **CAS 54711-39-6**. U(eq) is defined as one third of the trace of the orthogonalized U^{ij} tensor.

	x	y	z	U(eq)
O(1)	7678(1)	2102(1)	5283(1)	17(1)
O(2)	9492(1)	1534(1)	6716(1)	22(1)
O(3)	4567(1)	2279(1)	8813(1)	22(1)
N(1)	4254(2)	2901(2)	2048(1)	16(1)
C(1)	7812(2)	1880(2)	6382(1)	16(1)
C(2)	5942(2)	2097(2)	7017(1)	15(1)
C(3)	4162(2)	2389(2)	6530(1)	15(1)
C(4)	4091(2)	2528(2)	5407(1)	14(1)
C(5)	2340(2)	2783(2)	4842(1)	16(1)
C(6)	2380(2)	2924(2)	3750(1)	17(1)
C(7)	4214(2)	2792(2)	3136(1)	15(1)
C(8)	5982(2)	2521(2)	3694(1)	15(1)
C(9)	5888(2)	2399(2)	4793(1)	14(1)
C(10)	6052(2)	1961(2)	8182(1)	17(1)
C(11)	2496(2)	2993(2)	1456(1)	18(1)
C(12)	898(2)	5118(2)	1315(1)	23(1)
C(13)	6027(2)	3006(2)	1405(1)	17(1)

Table S3. Atomic coordinates (x 10^4) and equivalent isotropic displacement parameters ($Å^2$x 10^3) for Compound **3**. U(eq) is defined as one third of the trace of the orthogonalized U^{ij} tensor.

	x	y	z	U(eq)
O(1)	2633(2)	4804(1)	6469(1)	18(1)
O(2)	1889(2)	6631(1)	7941(1)	24(1)
O(3)	8166(2)	6926(1)	10682(1)	24(1)
N(1)	3858(2)	1007(2)	3076(2)	20(1)
C(1)	3298(3)	5813(2)	7689(2)	18(1)
C(2)	5621(3)	5789(2)	8540(2)	17(1)
C(3)	7070(3)	4836(2)	8100(2)	18(1)
C(4)	6358(3)	3840(2)	6838(2)	17(1)
C(5)	7767(3)	2846(2)	6302(2)	18(1)
C(6)	6954(3)	1913(2)	5067(2)	17(1)
C(7)	4640(3)	1919(2)	4291(2)	17(1)

C(8)	3201(3)	2901(2)	4798(2)	18(1)
C(9)	4080(3)	3834(2)	6036(2)	16(1)
C(10)	6286(3)	6812(2)	9854(2)	20(1)
C(11)	4940(3)	-56(2)	2259(2)	19(1)
C(12)	2613(3)	-380(2)	1151(2)	19(1)
C(13)	1586(3)	709(2)	2118(2)	19(1)

Numbering for CAS 54711-39-6 (**Table S2**)

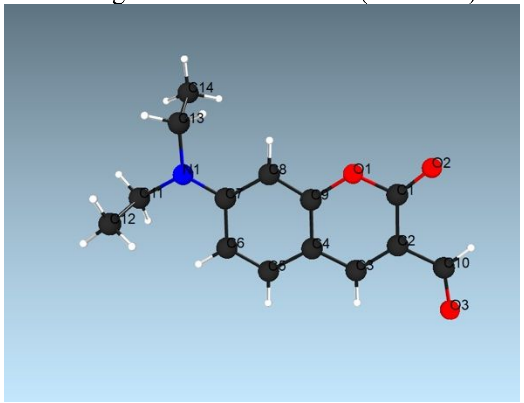

Numbering for **3 (Table S3)**

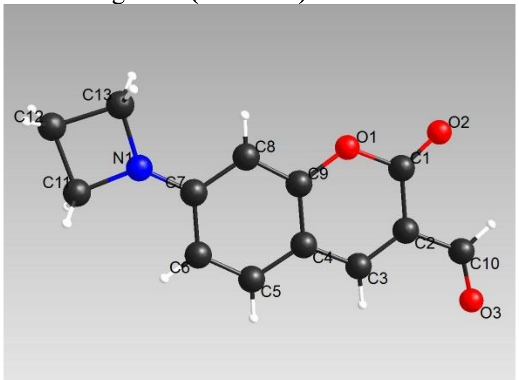

Supplement: Supplementary file 1 [file bi4c00750_si_001.pdf]
